# Opportunities and challenges with artificial intelligence in allergy and immunology: a bibliometric study

**DOI:** 10.3389/fmed.2025.1523902

**Published:** 2025-04-09

**Authors:** Ningkun Xiao, Xinlin Huang, Yujun Wu, Baoheng Li, Wanli Zang, Khyber Shinwari, Irina A. Tuzankina, Valery A. Chereshnev, Guojun Liu

**Affiliations:** ^1^Department of Immunochemistry, Institution of Chemical Engineering, Ural Federal University, Yekaterinburg, Russia; ^2^Laboratory for Brain and Neurocognitive Development, Department of Psychology, Institution of Humanities, Ural Federal University, Yekaterinburg, Russia; ^3^Preventive Medicine and Software Engineering, West China School of Public Health, Sichuan University, Chengdu, China; ^4^Engineering School of Information Technologies, Telecommunications and Control Systems, Ural Federal University, Yekaterinburg, Russia; ^5^Postgraduate School, University of Harbin Sport, Harbin, China; ^6^Laboratório de Biologia Molecular de Microrganismos, Universidade São Francisco, Bragança Paulista, Brazil; ^7^Department of Biology, Nangrahar University, Nangrahar, Afghanistan; ^8^Institute of Immunology and Physiology of the Ural Branch of the Russian Academy of Sciences, Yekaterinburg, Russia; ^9^School of Life Science and Technology, Inner Mongolia University of Science and Technology, Baotou, China

**Keywords:** artificial intelligence, allergy and immunology, immunology, machine learning, deep learning, health management, bibliometric study

## Abstract

**Introduction:**

The fields of allergy and immunology are increasingly recognizing the transformative potential of artificial intelligence (AI). Its adoption is reshaping research directions, clinical practices, and healthcare systems. However, a systematic overview identifying current statuses, emerging trends, and future research hotspots is lacking.

**Methods:**

This study applied bibliometric analysis methods to systematically evaluate the global research landscape of AI applications in allergy and immunology. Data from 3,883 articles published by 21,552 authors across 1,247 journals were collected and analyzed to identify leading contributors, prevalent research themes, and collaboration patterns.

**Results:**

Analysis revealed that the USA and China are currently leading in research output and scientific impact in this domain. AI methodologies, especially machine learning (ML) and deep learning (DL), are predominantly applied in drug discovery and development, disease classification and prediction, immune response modeling, clinical decision support, diagnostics, healthcare system digitalization, and medical education. Emerging trends indicate significant movement toward personalized medical systems integration.

**Discussion:**

The findings demonstrate the dynamic evolution of AI in allergy and immunology, highlighting the broadening scope from basic diagnostics to comprehensive personalized healthcare systems. Despite advancements, critical challenges persist, including technological limitations, ethical concerns, and regulatory frameworks that could potentially hinder further implementation and integration.

**Conclusion:**

AI holds considerable promise for advancing allergy and immunology globally by enhancing healthcare precision, efficiency, and accessibility. Addressing existing technological, ethical, and regulatory challenges will be crucial to fully realizing its potential, ultimately improving global health outcomes and patient well-being.

## Highlights

- Artificial intelligence (AI) is increasingly used in allergy and immunology, particularly machine learning (ML) and deep learning, with a trend towards integration with personalized medicine.
- AI models demonstrate rapid and precise processing of high-dimensional and complex data, potentially accelerating disease classification, prediction, regulation, and treatment in allergy and immunology.
- Countries like the USA and China hold significant leading positions in the field, with trends suggesting an expansion of their leadership.
- Challenges in AI within this field focus on model interpretability, fairness, robustness, the legality of data sources, and the integrity of regulatory systems.

## 1 Introduction

In recent years, the healthcare sector has witnessed a surge in data production and a substantial increase in medical costs, propelling artificial intelligence (AI) to the forefront of healthcare innovation. AI profoundly impacts disease prediction and prevention, patient care, diagnostics, management, drug development, and the operation of smart hospital systems (1–3). AI in healthcare employs algorithms and software to simulate human cognition in the analysis of complex medical data, undertaking tasks such as learning, language processing, knowledge synthesis, reasoning, and self-correction (1). Machine learning (ML) and deep learning (DL), in particular, have seen expansive applications in the current healthcare landscape. ML, a subset of AI, excels at enabling systems to learn from data and improve from experience without explicit programming (4). This aligns with the healthcare sector’s growing need to process various types of data, such as imaging and audiovisual information. ML algorithms can rapidly and precisely process these data types, unraveling complex relationships and learning to perform specific tasks, thus aiding clinicians in diagnosis, disease prediction, and recommending treatment modalities. DL, a specialized subset of ML, mimics the human brain’s data processing through neural network layers (5), demonstrating significant efficacy in recognizing patterns and features within large, complex datasets. This includes medical imaging, video, speech processing (5–7), predicting drug activities (8), identifying DNA sequence patterns, and forecasting genetic disorders and susceptibility to certain diseases (9).

Currently, AI is widely applied in oncology, cardiovascular diseases, and radiology (2, 10), with increasing applications in the fields of allergy and immunology. A lot of high-dimensional complex data is created in the field of allergy and immunology. AI makes it easier to organize and look for patterns in clinical symptoms, environmental data, and genetic information. This aids in more accurate diagnoses of allergens and autoimmune diseases (11), enhances the efficacy and specificity of clinical decision-making and practice, dynamically adjusts treatment recommendations (12), predicts drug development outcomes and adverse drug reactions, and identifies patients at higher risk for allergies (13, 14). AI swiftly analyzes intricate immunological datasets, propelling rapid advancements in genomics, proteomics, and other omics technologies. This accelerates the identification of biomarkers and mechanistic pathways involved in immune responses (15), establishes digital systems, streamlines practice management processes, and increases the efficiency of medical practice management (16).

As the application of AI in allergy and immunology continues to expand, systematic analysis of global research hotspots and trends in this field becomes increasingly important. However, there is currently a lack of bibliometric studies exploring this domain. Bibliometrics utilizes mathematical and statistical methods to quantitatively analyze and integrate global research progress and trends with visualization techniques, assisting researchers in quickly grasping current hotspots and frontiers, consolidating research information, and fostering communication and collaboration among researchers (17, 18). Therefore, we employed bibliometric methods to conduct a comprehensive analysis of AI-related articles published in the field of allergy and immunology, enhancing our understanding of the domain.

## 2 Methods

### 2.1 Literature search and data collection

**Figure 1:**
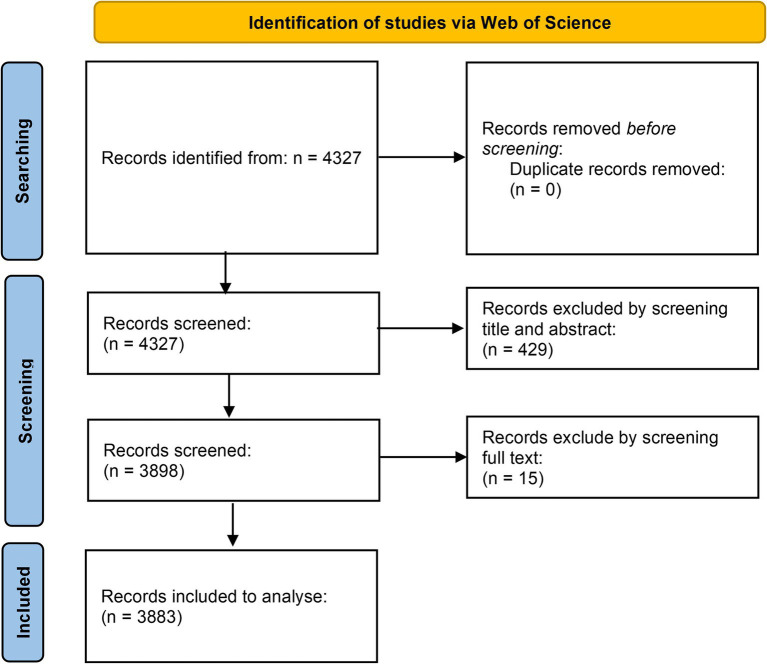
Flow chart of included and excluded studies.

We conducted our analysis using the Web of Science Core Collection (WoSCC): Science Citation Index Expanded (SCIE) and Social Sciences Citation Index (SSCI) as our data source. We ensured the validity of our conclusions by using the WoSCC, a globally recognized authoritative database and the most utilized resource in bibliometric research (19). Our search strategy encompassed two thematic areas: keywords relating to AI, allergies, and immunology, along with their synonyms. The search strategy was as follows: TS = (“Allergy” OR “Immunology” OR “Clinical Immunology” OR “Immunochemistry” OR “Immunoinformatics” OR “immunolog*” OR “Allergy Specialty” OR “Allergic Reactions” OR “Hypersensitivity” OR “Immune System” OR “Allergy” OR “Food Allergy” OR “Asthma” OR “Bronchial Asthma” OR “Primary immunodeficiency” OR “Primary Immune Deficiency” OR “Autoimmune Diseases” OR “Anaphylaxis” OR “Atopic Dermatitis” OR “Eosinophilic Disorders” OR “Immunodeficiency Syndromes” OR “Allergen-specific Immunotherapy”) AND TS = (“artificial intelligence” OR “computational intelligence” OR “machine learning” OR “deep learning” OR “neural networks” OR “reinforcement learning” OR “random forest” OR “support vector machine” OR “big data” OR “image processing” OR “feature learning” OR “convolutional neural networks” OR “evolutionary algorithms” OR “natural language processing” OR “Bayesian networks” OR “Bayesian learning” OR “multiagent systems”), with the search culminating on August 15, 2024. We limited the inclusion criteria to articles and reviews published in English. Two authors reviewed the titles and abstracts of 4,327 papers from WOSCC. Among the 4,346 papers retrieved from WoSCC, we excluded 432 articles belonging to the fields of information security, architecture, materials science, landscape architecture, botany, neurology, dermatology, cardiology, and food science, and excluded 12 retracted articles, yielding 3,883 papers (see Figure 1 for details). Out of 3,883 papers, 511 were reviews and 3,372 were original articles. Furthermore, of the 3,372 original articles included, 296 referenced or employed bioinformatics methodologies. Then we exported the selected publications in plain text and table-delimited formats, capturing essential details such as publication year, title, authors, abstracts, and keywords.

### 2.2 Data analysis

We imported the data into CiteSpace V5.7.R1 (Drexel University, Chaomei Chen, USA) and Excel 2019 (Microsoft, Washington, United States) for deduplication, organization, and analysis. No duplicate articles were found. CiteSpace, a bibliometric program developed by Chaomei Chen based on JAVA, is widely used in the field of bibliometrics (18, 20). We also utilized https://bibliometric.com/ and the bibliometrix package in R software version 4.0.3 to analyze collaborative relationships between different authors, countries, and regions. In the presented visualizations, the weight of the parameters determines the size of the nodes, the more significant the weight, the larger the node. Each node represents different parameters, including countries, institutions, authors, and keywords. We assign different colors to nodes and lines based on their cluster type or timeline, with identical colors denoting the same cluster or timeline. The thickness of the connecting lines indicates the strength of the linkage (17).

## 3 Results and discussion

### 3.1 Publication volume and growth trend

**Figure 2:**
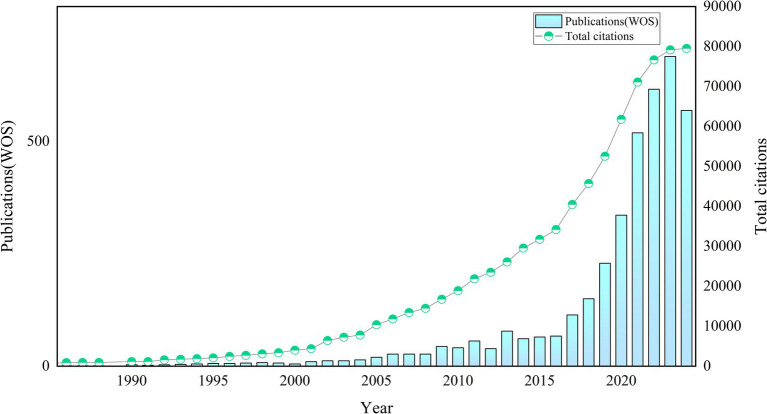
Trends of annual publications on AI in allergy and immunology.

As of August 15, 2024, we had included 3,883 relevant publications in the WoSCC. Figure 2 illustrates the publication trends of AI-related literature in allergy and immunology. Prior to 2001, the growth of publications in this domain was slow. Between 2002 and 2015, the number of publications began to increase, with rapid growth observed after 2015. The field has experienced vigorous development in recent years, as evidenced by the publication of 2,958 articles over the past 6 years (2019–2024), accounting for 76.18% of the total output. Citation data show a rise in citations starting in 2002, with particularly sharp increases after 2016. This positive momentum has persisted in recent years. The 3,883 relevant publications had received 79,539 citations as of August 15, 2024, with an average of 20.5 citations per article (see Figure 2 for details).

### 3.2 Country/region, and institutional analysis

**Figure 3:**
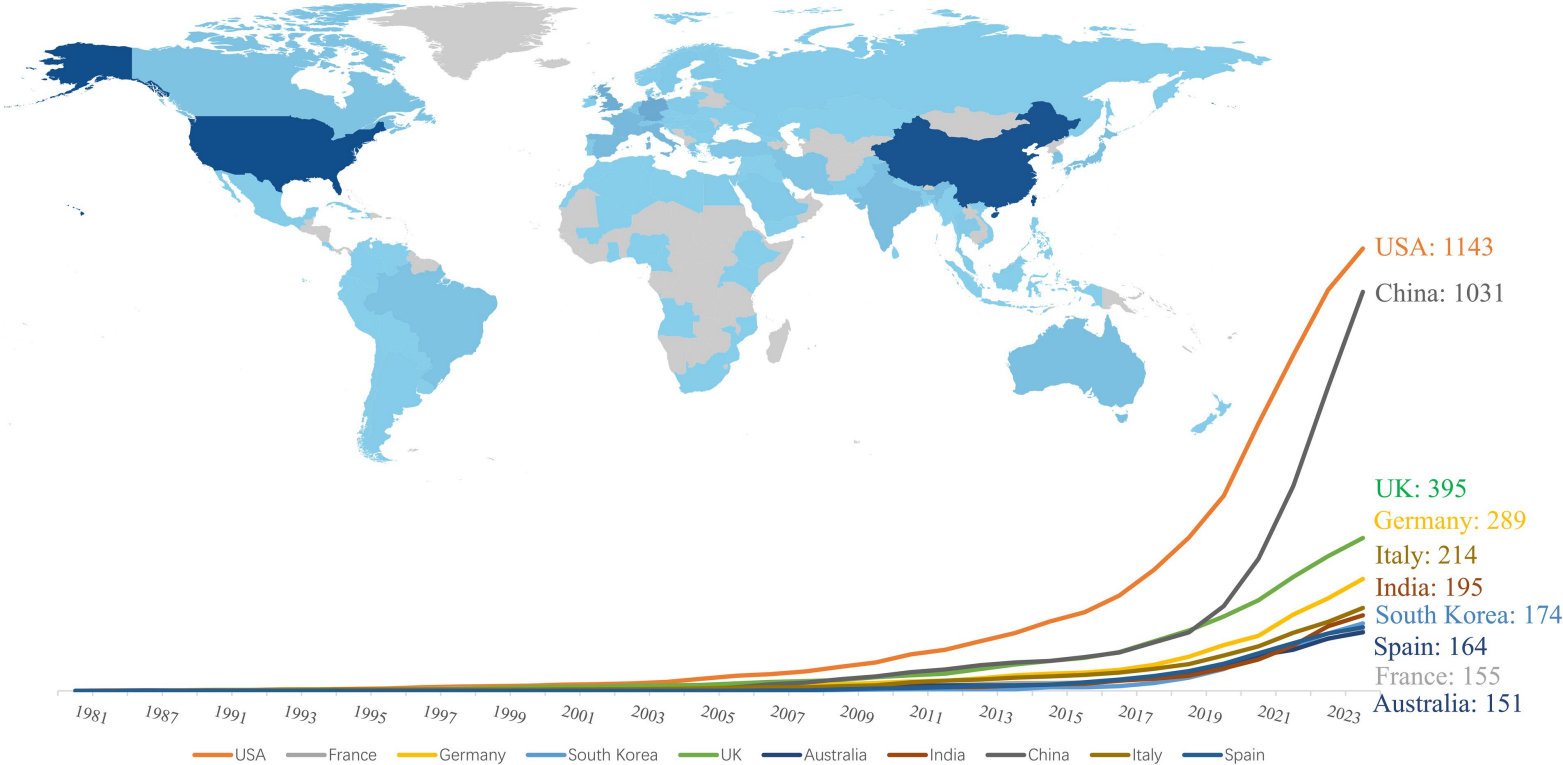
Top 10 countries with highest publications on AI in allergy and immunology. (i) By bibliometrix; (ii) darker colors in the graph represent a higher number of publication outputs.

**Figure 4:**
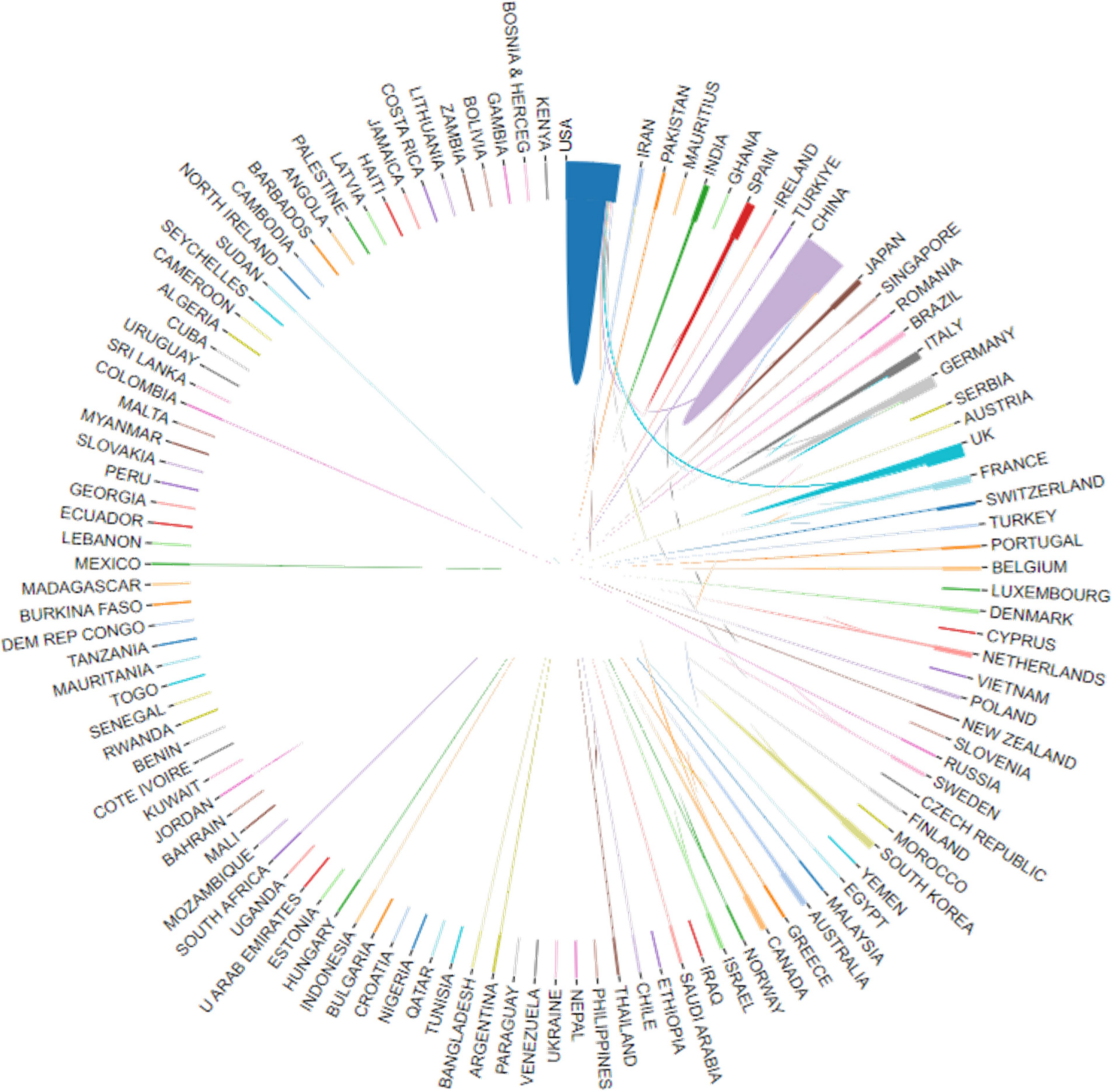
The international collaboration visualization map on AI in allergy and immunology. (i) By website (https://bibliometric.com/app_v0); (ii) the thicker the line, the closer the cooperation between countries.

Researchers from 113 countries and regions have contributed to the study of AI in allergy and immunology, leading to numerous publications in this field. In 1981, the first article in this domain was published in Experimental Pathology by German scholar Haroske et al. (21). The article discussed the utilization of automated image processing to distinguish the subtle characteristics of various groupings of human lymphocytes in unpurified, unlabeled lymphocyte suspensions obtained from peripheral blood. Despite Germany’s early contribution, the USA has emerged as the leading country in terms of publication volume. Farmer et al. (22) authored the first USA publication in this field in Physica D: Nonlinear Phenomena, detailing a dynamic model of the immune system based on the Jerne network hypothesis. Since 2016, the USA has seen a rapid increase in related research output. Between 1986 and 2015, the USA published 179 articles in this field, but from 2016 to 2024, this number surged to 964, bringing the total to 1,143 articles, representing 29.44% of the global output. China, which published its first related research in 2001, has also seen significant growth, particularly after 2016, with 1,031 articles published by 2024, accounting for 26.55% of the global research output. The USA and China now dominate this field, publishing the largest number of articles and engaging in extensive collaborations with other countries, with strong growth momentum continuing (see Figures 3, 4). Among the top 10 countries contributing to publications, the UK has participated in 395 studies, followed by Germany in 289, Italy in 214, India in 195, South Korea in 174, Spain in 164, France in 155, and Australia in 151 studies. Moreover, Figure 4 illustrates that in addition to China and the USA, nations including the UK, Germany, France, Japan, and South Korea are also performing a large amount of collaborative research in this domain. This is due to these countries presently occupying favorable positions in vaccine development, clinical trials, and AI-enhanced immunology research.

We also analyzed the affiliations of the corresponding authors for the 3,883 articles. Among these, 961 articles (24.75% of the total) had corresponding authors from China, with 821 articles having Chinese authors as the sole corresponding authors and 140 articles co-authored with corresponding authors from other countries. The USA contributed 847 articles (21.81% of the total), with 629 of these having USA-based sole corresponding authors and 218 co-authored with corresponding authors from other countries. Together, China and the USA accounted for 46.56% of the corresponding authority in this field, indicating their significant leadership (see Table 1). Additionally, 214 articles from the UK, 159 from Germany, 153 from India, 139 from South Korea, 134 from Italy, 93 from Spain, 85 from France, and 83 from Australia represented corresponding authorship.

**Table 1:** Top 10 countries with corresponding authors of publications on AI in allergy and immunology.

Country	Publications	SCP	MCP	Frequency	MCP_Ratio
China	961	821	140	0.247	0.146
USA	847	629	218	0.218	0.257
UK	214	117	97	0.055	0.453
Germany	159	87	72	0.041	0.453
India	153	114	39	0.039	0.255
South Korea	139	105	34	0.036	0.245
Italy	134	91	43	0.035	0.321
Spain	93	50	43	0.024	0.462
France	85	39	46	0.022	0.541
Australia	83	47	36	0.021	0.434

SCP, Single country publications; MCP, Multiple country publications; Frequency, Number of publications in this country/total number of publications; MCP_Ratio, MCP/Publications.

**Figure 5:**
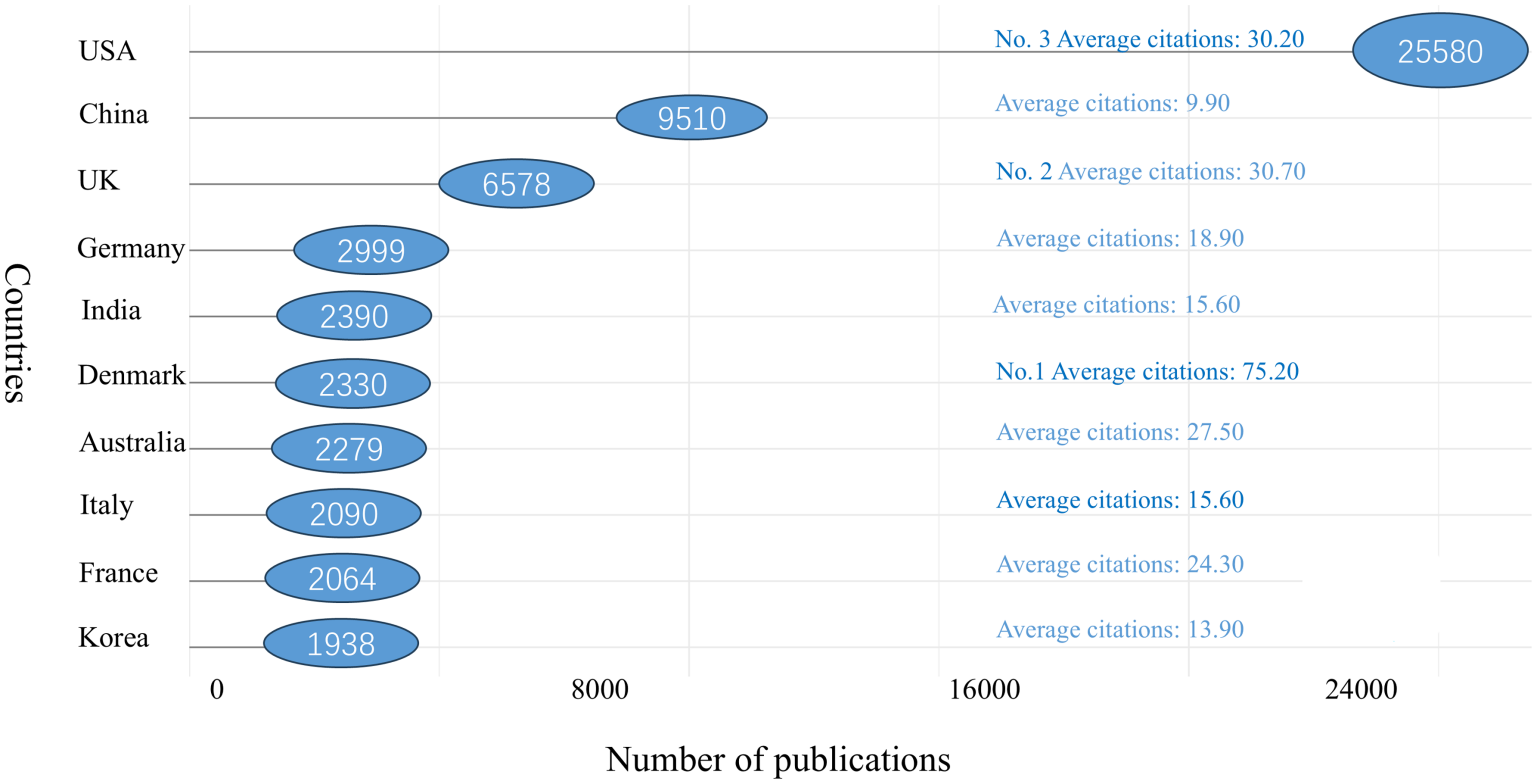
Top 10 countries with highest citations on AI in allergy and immunology.

In terms of citation counts, the USA leads with 25,580 citations, followed by China with 9,510 citations. Other top 10 countries by citation count include the UK (*n* = 6,578), Germany (*n* = 2,999), India (*n* = 2,390), Denmark (*n* = 2,330), Australia (*n* = 2,279), Italy (*n* = 2,090), France (*n* = 2,064), and South Korea (*n* = 1,938). Denmark ranks first in average citations per paper, with an impressive 75.20 citations per article. The UK follows with 30.70 citations per article, with the USA close behind at 30.20 citations per article. China, however, lags with an average of only 9.90 citations per article, indicating that it still has some ground to cover in terms of impact (see Figure 5 for details).

**Figure 6:**
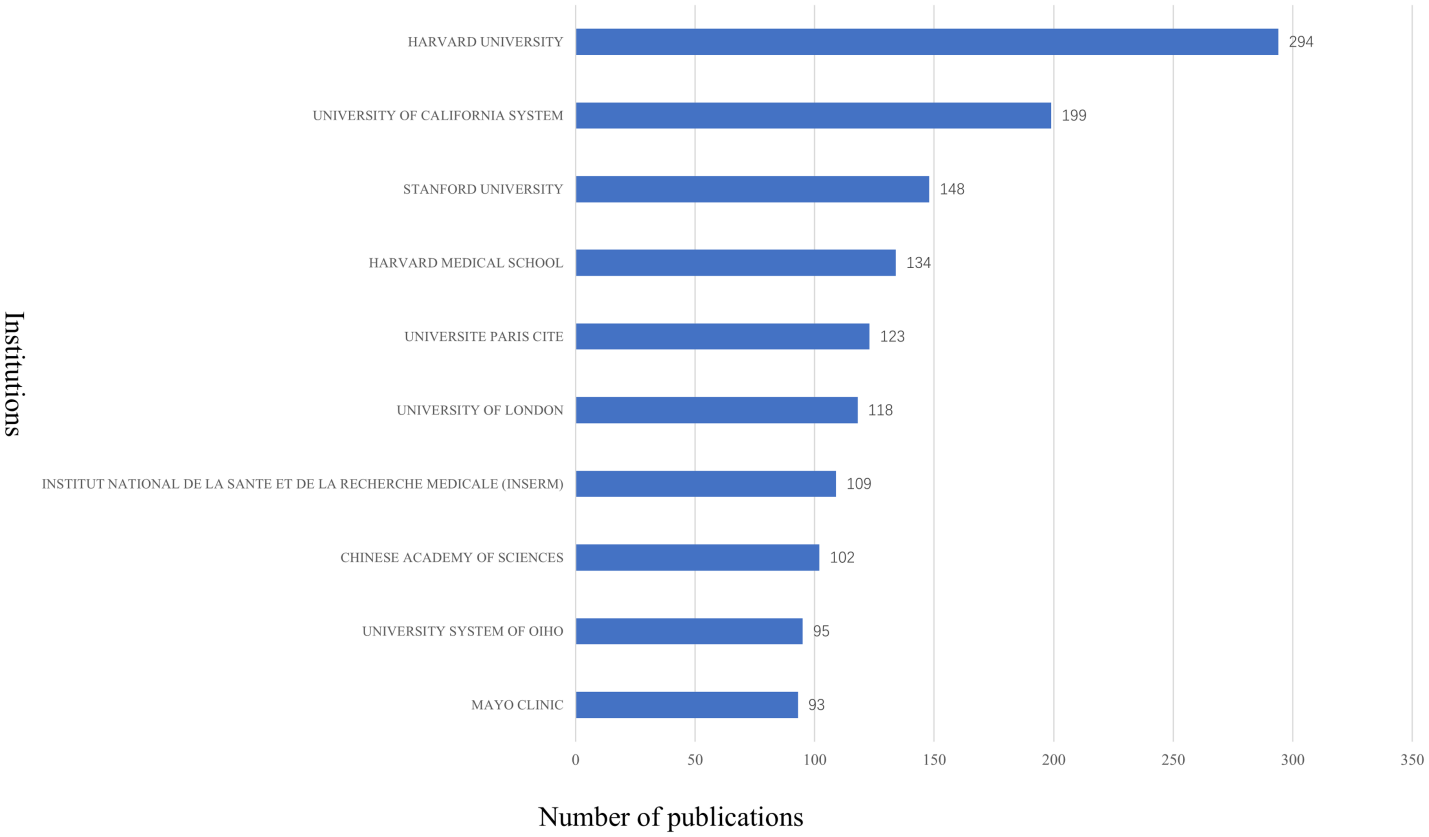
Top 10 institutions with highest publications on AI in allergy and immunology.

A total of 4,269 institutions contributed to the 3,883 published articles. Harvard University leads the field with 294 publications, followed by the University of California system with 199 articles, and Stanford University with 148 articles. Other top institutions include Harvard Medical School (134 articles), Université Paris Cité (123 articles), University of London (118 articles), Institut National de la Santé et de la Recherche Médicale (109 articles), Chinese Academy of Sciences (102 articles), University of Ohio (95 articles), and Mayo Clinic (93 articles) (see Figure 6 for details). Of the top 10 institutions, six are based in the USA, two in France, one in China, and one in the UK, further underscoring the USA’s significant dominance in this field.

In conclusion, the USA and China dominate allergology and immunology research, with the USA leading in impact and China excelling in publication volume but lagging in influence. The USA’s strong research infrastructure, high international collaboration, and leadership in pharmaceuticals and AI-driven immunology contribute to its global authority. In contrast, China’s lower international collaboration limits its influence, despite significant government investment and vaccine advancements.

European nations-France, Germany, Spain, and the UK-stand out for their high international collaboration, which enhances research impact. Meanwhile, South Korea and India are emerging players but require greater global integration to improve their influence. Strengthening international partnerships will be key for China and other rising nations to match the USA’s research impact in the future.

### 3.3 Author analysis

A total of 21,552 authors have contributed to research on AI in allergy and immunology. We have summarized and analyzed the top 15fifteen most productive authors (see Table 2 for details). Adnan Custovic from Imperial College London leads the field with 34 related publications between 2010 and 2024. His main research interests are using ML to assess the types of asthma and eczema people will have (23, 24), figuring out how allergen microarrays relate to clinical symptoms (25), and studying how allergic sensitization happens and what factors make it more likely to happen (26–28). He has also worked on developing predictive models for asthma in children and adults (29), as well as investigating the application of AI in allergy research (30, 31). Morten Nielsen, from the Technical University of Denmark, published 21 related publications from 2005 to 2024. His research interests include using ML to improve prediction algorithms for MHC Class I/II, T cells, and B cell epitopes (32–34), enhancing understanding of cellular immunopeptides (35), and improving predictions of protein drug immunogenicity (36). Angela Simpson from the University of Manchester has closely collaborated with Adnan Custovic, publishing 21 papers between 2010 and 2024. She likes to use AI to look into different sensitization patterns related to asthma (28), study the link between different allergic phenotypes and asthma (37), find asthma subgroups (38), predict phenotypes for asthma and eczema (24), and look into the relationship between cytokine expression patterns and clinical outcomes (39, 40). Gang Luo, from the University of Washington in the USA, published 14 related articles between 2015 and 2024. He has extensively worked on using AI to predict childhood asthma (41, 42), assist asthma patients in seeking medical care (43), and support clinical decision-making for physicians (44). Tesfaye B. Mersha from the University of Cincinnati published 13 related papers between 2015 and 2024. His research focuses on using ML and deep learning for multi-omics analysis (45), analyzing sex dimorphism (46), predicting individual risk genes, and promoting the development of precision medicine (47). He has also developed and validated risk prediction models for asthma (48).

**Table 2:** Top 15 productive authors with publications on AI in allergy and immunology.

Rank	Author	Current affiliations and countries	Number of publications	Local citations
1	Adnan Custovic	Imperial College London, UK	34	263
2	Morten Nielsen	Technical University of Denmark, Denmark	21	161
2	Angela Simpson	University of Manchester, UK	21	178
4	Gang Luo	University of Washington, USA	14	77
5	Tesfaye B. Mersha	University of Cincinnati, USA	13	36
6	Kimberly G. Blumenthal	Massachusetts General Hospital, USA	12	21
6	Yaron Ilan	Hebrew University, Israel	12	57
6	Bjoern Peters	La Jolla Institute for Allergy and Immunology, USA	12	148
6	Gajendra P.S. Raghava	Indraprastha Institute of Information Technology, India	12	58
6	Scott T. Weiss	Harvard Medical School, USA	12	36
11	Yudong Cai	Shanghai University, China	11	15
12	Salih Güneş	Selcuk University, Turkey	10	10
12	Kemal Polat	Bolu Abant İzzet Baysal Üniversitesi, Turkey	10	38
12	Nima Aghaeepour	Stanford University School of Medicine, USA	10	16
12	Vladimir Brusic	University of Nottingham Ningbo, China	10	21

Kimberly G. Blumenthal from Massachusetts General Hospital, Bjoern Peters from the La Jolla Institute for Allergy and Immunology, and Scott T. Weiss from Harvard Medical School, along with Yaron Ilan from Hebrew University in Israel and Gajendra P.S. Raghava from the Indraprastha Institute of Information Technology in India, each published 12 articles in this field. Kimberly G (see Table 2 for details). Blumenthal began her research in 2016, focusing on using ML to explore new approaches to studying the epidemiology of drug allergies (49), improving methods in electronic health records (EHRs) (50, 51), and developing models for predicting patient allergic reactions (52). Yaron Ilan published 12 articles from 2021 to 2024, advocating for and advancing the development of second-generation AI systems to improve diagnosis and detection of rare diseases (53), optimize drug dosing and study drug resistance (54, 55), and enhance global healthcare in an intelligent, digital manner to reduce healthcare costs (56). Bjoern Peters has established a strong collaboration with Morten Nielsen. In 2007, Bjoern Peters published his first article in this field, aligning his research interests with Nielsen’s. He focuses on using ML to predict the binding of peptides to MHC Class I/II molecules and exploring non-canonical patterns (57, 58). Peters has also worked on improving predictions for MHC II antigen presentation (59), as well as T cell and B cell epitope prediction (60, 61). From 2007 to 2024, Gajendra P.S. Raghava published 11 related papers, optimizing methods for predicting linear and conformational B cell epitopes in antigens (62), and improving algorithms for predicting and identifying receptors and defensins (63, 64). Scott T. Weiss studies how ML can be used to evaluate how people will react to short-acting bronchodilators (65), figure out gene expression and genetic polymorphisms (66), look into the molecular mechanisms of asthma (67), investigate childhood asthma metabolomics (68) and develop new models for using electronic health records to test and predict asthma (69).

Among other prolific contributors, Yudong Cai from Shanghai University published 11 related papers between 2011 and 2024, with a focus on using ML to identify immune gene signatures of immune cell subtypes (70), particularly in identifying immune cell markers for COVID-19 (71, 72). Salih Güneş from Selcuk University published 10 relevant publications from 2005 to 2013. Kemal Polat from Bolu Abant İzzet Baysal University, who collaborated extensively with Salih Güneş, published 10 publications between 2004 and 2009. Their research primarily explored computer-assisted medical diagnostic systems and medical decision support systems supported by algorithms like the artificial immune recognition system (AIRS), fuzzy weighted pre-processing, feature selection, and component analysis (73, 74). Nima Aghaeepour from Stanford University School of Medicine also published 10 related papers, primarily exploring the relationships between patient emotions, lifestyle, and peripheral and systemic immune responses using ML and deep learning (75, 76). Vladimir Brusic from the University of Nottingham Ningbo in China published 10 related articles between 2002 and 2022, focusing on using ML to develop new tools for analyzing immunological data (77, 78).

Among these prolific authors, Adnan Custovic ranks first in local citations with 263, followed by Angela Simpson with 178 citations and Morten Nielsen with 161 (see Table 2 for details). These highly productive and highly cited authors have contributed to varying extents to the practical application of AI in this field.

### 3.4 Journal and publication analysis

We dispersed publications related to AI in allergy and immunology across 1,247 journals. We classified these journals according to Bradford’s Law, which divides journals into three zones, each containing approximately one-third of the total publications. This classification helps identify the most influential core journals within a specific academic field (19, 79). Our analysis revealed 42 journals in Zone 1, 233 in Zone 2, and 972 in Zone 3. We summarized the impact factor (IF), quartile, and category for the top 15 journals in Zone 1 (see Table 3 for details). Frontiers in Immunology emerged as the leading journal in this field, publishing 199 papers, which account for 5.12% of the domain’s output. This was followed by Scientific Reports with 90 publications and PLOS One with 69 publications. Other journals in the top 15 include IEEE Access (*n* = 49), Journal of Allergy and Clinical Immunology (*n* = 48), BMC Bioinformatics (*n* = 46), Briefings in Bioinformatics (*n* = 46), Frontiers in Genetics (*n* = 45), Bioinformatics (*n* = 35), International journal of Molecular Sciences (*n* = 35), Allergy (*n* = 35), Cancers (*n* = 29), Computers in Biology and Medicine (*n* = 29), Heliyon (*n* = 29), and Journal of Allergy and Clinical Immunology-in Practice (*n* = 29). Together, the top 15 journals published 807 papers, accounting for 20.78% of the total publications in the field.

**Table 3:** Top 15 journals by number of publications on AI in allergy and immunology.

Rank	Journal	Publications (%)^a^	cumPub (%)^b^	IF (202 3)^c^	JCR Category (SCIE)^d^
1	Frontiers in Immunology	199 (5.12%)	199 (5.12%)	5.7, Q1	Immunology
2	Scientific Reports	90 (2.32%)	289 (7.44%)	3.8, Q1	Multidisciplinary Sciences
3	Plos One	69 (1.78%)	358 (9.22%)	2.9, Q1	Multidisciplinary Sciences
4	Ieee Access	49 (1.26%)	407 (10.48%)	3.4, Q2	Computer Science, Information Systems; Engineering, Electrical & Electronic; Telecommunications
5	Journal of Allergy and Clinical Immunology	48 (1.24%)	455 (11.72%)	11.4, Q1	Allergy; Immunology
6	BMC Bioinformatics	46 (1.18%)	501 (12.90%)	2.9, Q2	Biochemical Research Methods; Biotechnology & Applied Microbiology; Mathematical & Computational Biology
7	Briefings in Bioinformatics	46 (1.18%)	547 (14.09%)	6.8, Q1	Biochemical Research Methods; Mathematical & Computational Biology
8	Frontiers in Genetics	45 (1.16%)	592 (15.25%)	2.8, Q2	Genetics & Heredity
9	Bioinformatics	35 (0.90%)	627 (16.15%)	4.4, Q1	Biochemical Research Methods; Biotechnology & Applied Microbiology; Mathematical & Computational Biology
10	International Journal of Molecular Sciences	35 (0.90%)	662 (17.05%)	4.9, Q1	Biochemistry & Molecular biology; Chemistry, Multidisciplinary
11	Allergy	29 (0.75%)	691 (17.80%)	12.6, Q1	Allergy; Immunology
12	Cancers	29 (0.75%)	720 (18.54%)	4.5, Q1	Oncology
13	Computers in Biology and Medicine	29 (0.75%)	749 (19.29%)	7.0, Q1	Computer Science, Interdisciplinary applications; Engineering, Biomedical; Biology; Mathematical & Computational Biology
14	Heliyon	29 (0.75%)	778 (20.04%)	3.4, Q1	Multidisciplinary Sciences
15	Journal of Allergy and Clinical Immunology-in Practice	29 (0.75%)	807 (20.78%)	8.2, Q1	Allergy; Immunology

a Percentage of total published articles in the journal.

b Cumulative publications as a percentage of total publications.

c Journal impact factor (Journal Citation Reports 2023).

d SCIE, Science Citation Index Expanded.

An analysis of local citations within the field can provide insights into foundational research and emerging hotspots. We ranked the local citations of the 3,883 included publications and identified the top 15 most-cited articles (see Table 4 for details). The most locally cited article, authored by Vanessa Jurtz in 2017 with 44 local citations, introduced NetMHCpan-4.0, a novel ML framework trained to predict MHC peptide presentation. This method demonstrated strong predictive performance in identifying validated eluted ligand data, cancer neoantigens, and T cell epitopes (58). Joseph Finkelstein published the second most locally cited article in 2017 with 41 local citations, using ML to predict asthma exacerbations early. This highlights the significant potential of ML in developing remote monitoring systems for chronic diseases (80). The third-ranking article, with 35 local citations, was authored by Ilka Hoof in 2009 and focused on predicting peptide binding to MHC Class I molecules using NetMHCpan-2.0 (81). Martin Closter Jespersen and colleagues introduced the BepiPred-2.0 tool in 2017 (82), which garnered 32 local citations and ranked fourth. Bioinformatics widely used BepiPred-2.0 for predicting B cell epitopes in antigen sequences (82). Six of the top 15 most locally cited articles used ML to identify various asthma sensitization patterns, distinguish and predict asthma phenotypes, and develop allergen prediction models (28, 37, 80, 83–85). Four articles focused on ML algorithms to identify T and B cell epitope specificity, optimize antigen specificity recognition, and predict peptide binding to MHC Class I molecules (58, 81, 82, 86). Three articles summarized applications and developments of AL in the field of asthma (1, 87, 88). Additionally, two articles discussed dynamic models of the immune system and artificial immune systems (AIS) (22, 89).

**Table 4:** Top 15 most locally cited publications on AI in allergy and immunology.

Rank	Title, DOI	First author	Year	Journal	Local citations
1	NetMHCpan-4.0: Improved Peptide–MHC Class I Interaction Predictions Integrating Eluted Ligand and Peptide Binding Affinity Data, 10.4049/jimmunol.1700893.	Vanessa Jurtz	2017	The Journal of Immunology	44
2	Machine learning approaches to personalize early prediction of asthma exacerbations, 10.1111/nyas.13218.	Joseph Finkelstein	2017	Annals of the New York Academy of Sciences	41
3	NetMHCpan, a method for MHC class I binding prediction beyond humans, 10.1007/s00251-008-0341-z.	Ilka Hoof	2009	Immunogenetics	35
4	BepiPred-2.0: improving sequence-based B-cell epitope prediction using conformational epitopes, 10.1093/nar/gkx346.	Martin Closter Jespersen	2017	Nucleic Acids Research	32
5	Beyond atopy: multiple patterns of sensitization in relation to asthma in a birth cohort study, 10.1164/rccm.200907-1101OC.	Simpson Angela	2010	American journal of respiratory and critical care medicine	28
6	Multiple atopy phenotypes and their associations with asthma: similar findings from two birth cohorts, 10.1111/all.12134.	N. Lazic, G. Roberts	2013	Allergy	28
7	The immune system, adaptation, and machine learning, 10.1016/0167-2789(86)90240-X.	J. Doyne Farmer	1986	Physica D: Nonlinear Phenomena	27
8	Distinguishing Asthma Phenotypes Using Machine Learning Approaches, 10.1007/s11882-015-0542-0.	Rebecca Howard	2015	Current allergy and asthma reports	23
9	Artificial intelligence techniques in asthma: a systematic review and critical appraisal of the existing literature, 10.1183/13993003.00521-2020.	Konstantinos P. Exarchos	2020	European Respiratory Journal	23
10	AllerTOP v.2—a server for in silico prediction of allergens, 10.1007/s00894-014-2278-5.	Ivan Dimitrov	2014	Journal of molecular modeling	21
11	Artificial Intelligence/Machine Learning in Respiratory Medicine and Potential Role in Asthma and COPD Diagnosis, 10.1016/j.jaip.2021.02.014.	Alan Kaplan	2021	The Journal of Allergy and Clinical Immunology: In Practice	20
12	Developing a Model to Predict Hospital Encounters for Asthma in Asthmatic Patients: Secondary Analysis, 10.2196/16080.	Gang Luo	2020	JMIR medical informatics	19
13	Application of a Natural Language Processing Algorithm to Asthma Ascertainment. An Automated Chart Review, 10.1164/rccm.201610-2006OC.	Chung-Il Wi	2017	American journal of respiratory and critical care medicine	18
14	DeepTCR is a deep learning framework for revealing sequence concepts within T-cell repertoires, 10.1038/s41467-021-21879-w	John-William Sidhom	2021	Nature Communications	18
15	An artificial immune system for data analysis, 10.1016/S0303-2647(99)00092-1	Jon Timmis	2000	Biosystems	17

### 3.5 Keywords and hotspot analysis

**Figure 7:**
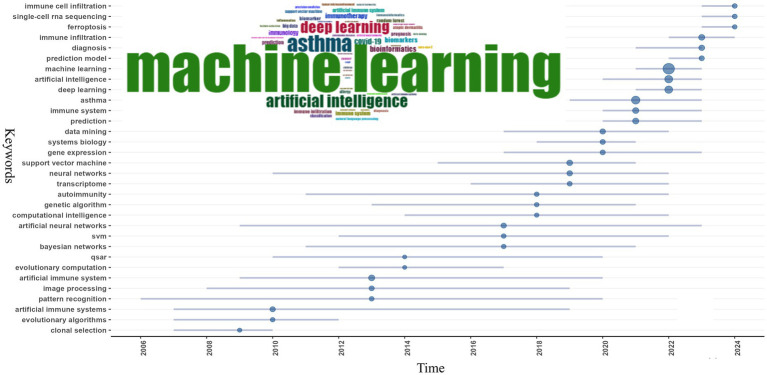
Word cloud and trend topics on AI in allergy and immunology.

Keyword co-occurrence analysis is instrumental in identifying focal points within a domain and exploring the internal connections among them. In the 3,883 publications included in our study authors used 8,849 distinct keywords. We selected the top 50 most frequent keywords for analysis. As shown in Figure 7 “machine learning” appeared most frequently with 871 mentions followed by “asthma” with 305 mentions “artificial intelligence” with 235 mentions and “deep learning” with 226 mentions. “AI” encompasses the broadest meaning among AI-related terms and ML is a subset of AI that involves algorithms learning from data to make predictions or decisions without explicit programming. Deep learning is a more complex subset of ML that uses neural networks with many layers (deep neural networks) to analyze various disease-related factors including genetic data and medical images. Overall the field often uses “artificial intelligence,” “machine learning,” and “deep learning” interchangeably despite their differing meanings. Other high-frequency keywords include “immune system” (*n* = 78) “artificial immune system” (*n* = 73) “prediction” (*n* = 73) “immune infiltration” (*n* = 69) “support vector machine” (*n* = 55) “diagnosis” (*n* = 53) “neural networks” (*n* = 42) and “data mining” (*n* = 40) (see Table 5 for details). The keywords “artificial immune system” and “immune system” reflect the symbiotic relationship between AI and immunology. Inspired by the biological immune system the AIS mimics its mechanisms to solve complex problems like pattern recognition anomaly detection predictive analysis and adaptive learning. In allergy and immunology AIS can simulate immune system responses under different conditions improving the accuracy of diagnosing allergic and immune diseases and facilitating personalized treatment plans based on individual immunological profiles (90). High-frequency terms like “prediction” and “diagnosis” highlight AI’s specific applications in the fields of immunology and allergy while the frequent mention of “asthma” underscores AI’s widespread application in asthma research. Indeed AI applications and advancements in asthma are the subject of nine out of the top 15 most locally cited articles (1, 28, 37, 80, 83–85, 87, 88).

**Table 5:** Commonly used AI terminology on AI in allergy and immunology.

Terminology	Simple definitions and applications
Machine Learning (ML)	A subset of AI that involves algorithms learning from data to make predictions or decisions without being explicitly programmed. In allergy and immunology, ML is used for predictive analytics, such as predicting allergic reactions or the outcomes of immunotherapies.
Deep Learning (DL)	A more complex subset of ML that uses neural networks with many layers (deep neural networks) to analyze various factors of diseases, including genetic data and medical images. It’s particularly effective in interpreting complex patterns like those found in high-dimensional data, essential for personalized medicine approaches in allergy and immunology.
Natural Language Processing (NLP)	A branch of AI that helps computers understand, interpret, and manipulate human language. NLP is applied in allergy and immunology to extract information from clinical notes and research papers, helping in the systematic review of literature and the extraction of patient information from electronic health records.
Swarm Learning (SL)	A novel approach that combines swarm intelligence with ML. SL can be particularly useful in allergy and immunology, for aggregating data across multiple sources without sharing the actual data, thus preserving privacy while enhancing predictive power.
Random Forests (RF)	An ensemble learning method that operates by constructing a multitude of decision trees at training time and outputting the class that is the mode of the classes of the individual trees. In allergy and immunology, RFs are used for classifying types of diseases and predicting patient responses to treatments based on genetic and environmental factors.
Support Vector Machines (SVM)	A supervised learning model that analyzes data for classification and regression analysis. SVMs are used in allergy and immunology to classify diseases and predict allergic reactions by learning hyperplanes that categorize new examples.
Convolutional Neural Networks (CNNs)	A type of deep neural network mostly used to analyze visual imagery. CNNs are employed in allergy and immunology for tasks like analyzing medical images, such as skin test reactions or cellular structures relevant to immunological research
Recurrent Neural Networks (RNNs)	Specialized RNNs effective in handling sequence data. They can model time-dependent data, making them suitable for tasks like modeling temporal immunological responses or predicting the progression of allergic diseases over time.
Graph Neural Networks (GNNs)	These networks extend DL techniques to graph data and can model relationships and interactions between entities (like cells or proteins). GNNs could be used in immunology to model complex interactions in the immune system or between allergens and antibodies.
Genetic Algorithms (GAs)	These are search-based algorithms inspired by the process of natural selection and genetics. In allergy and immunology, GAs can optimize complex problem-solving processes, such as tuning parameters in models predicting allergic reactions or optimizing treatment protocols based on genetic data.
Evolutionary Algorithms (EAs)	These algorithms use mechanisms inspired by biological evolution, such as reproduction, mutation, recombination, and selection. EAs can optimize the design of vaccines by finding the most effective combinations of antigens to elicit an immune response. They are also used in the optimization of treatment plans where multiple strategies might be tested to determine the best course of action for immunotherapy.
Transfer Learning	This technique involves taking a pre-trained model on one problem and reusing it on a second related problem. In allergy and immunology, transfer learning can be particularly effective when trained models from other medical domains are adapted to recognize or predict allergies and immune responses.
Reinforcement Learning (RL)	An area of ML concerned with how intelligent agents ought to take actions in an environment to maximize the notion of cumulative reward. RL can be used in therapeutic regimens where the treatment strategy is dynamically updated based on patient response.
Federated Learning	A ML technique that trains an algorithm across multiple decentralized devices or servers holding local data samples, without exchanging them. This approach is pertinent in allergy and immunology for collaborative studies across different regions or institutions while maintaining data privacy.
Feature Selection	This involves selecting the most relevant features from the data to improve model performance. In allergy and immunology, feature selection is crucial to identify the most relevant biomarkers or genetic variants that contribute to disease susceptibility and treatment outcomes.
Bayesian Networks	Probabilistic models that represent a set of variables and their conditional dependencies via a directed acyclic graph. In allergy and immunology, these networks are useful for disease risk assessment and understanding causal relationships between various biological factors.
Big Data	In allergy and immunology, big data encompasses vast amounts of information from clinical health records, immunological research data, genomic data, and more. By analyzing these large data sets, researchers and clinicians can identify trends in allergy and immune disease incidence, discover new biomarkers for diseases, and develop personalized medicine approaches based on patient genetics and environmental interactions.
Data Mining	Data mining involves exploring large databases to discover patterns and relationships within the data that can inform decisions and predict trends. This technique is used to sift through large amounts of medical and research data to find unexpected relationships and patterns that can lead to new hypotheses about immune responses or the development of allergies.
Explainable AI	Refers to methods and techniques in the application of AI such that the results of the solution can be understood by human experts. It bridges the gap between data scientists and domain experts in allergy and immunology, ensuring that the predictions and workings of AI models are transparent and understandable.

**Figure 8:**
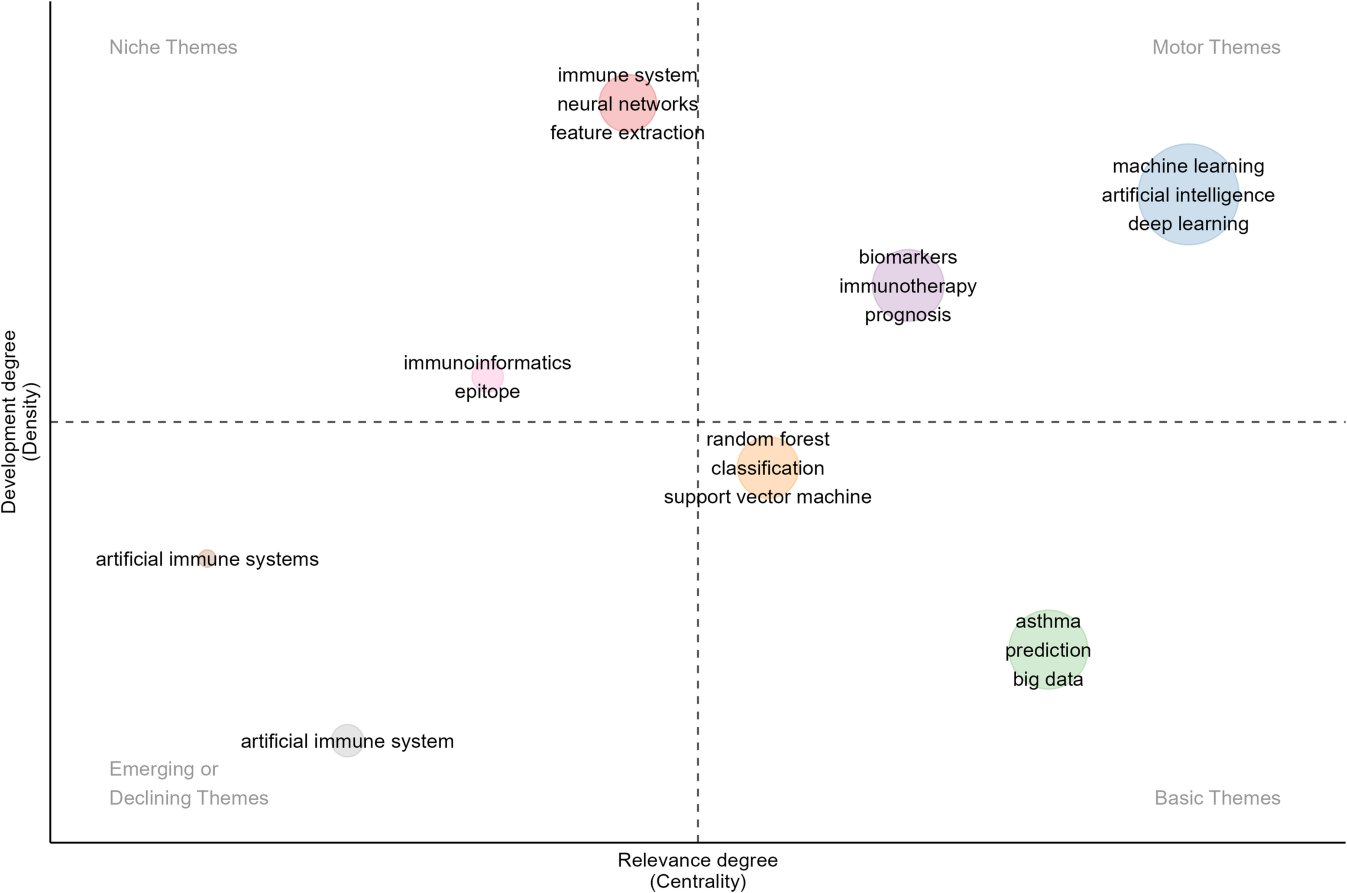
Thematic map on AI in allergy and immunology.

Additionally, we conducted a thematic map and evolutionary analysis of keywords to better understand the structure and dynamics within the research domain and predict future research focal points. As shown in Figure 8, the thematic map divides various topics into four quadrants based on centrality and density: (1) Upper Right—Motor Themes: This quadrant contains themes with high centrality and high density, indicating well-developed, mature, and reliable topics that are critical to the field. The themes of “machine learning,” “artificial intelligence,” “deep learning,” “biomarkers,” “immunotherapy,” and “prognosis” fall into this quadrant. From 2021 to 2023, ML and DL, as subsets of AI, have frequently appeared in immunology and allergy research. These technologies have applications in disease prediction, patient stratification, drug discovery, analysis of immune responses, development of personalized immunotherapies, clinical decision support, and prognosis management. Additionally, AI has made significant contributions to addressing the challenges posed by COVID-19, particularly in vaccine development, real-time tracking of viral mutations, and predicting patient outcomes. (2) Upper Left—Niche Themes: These themes exhibit low centrality but high density, indicating that they are well-researched yet have had less impact on the broader field. The themes of “immunoinformatics,” “epitope,” “immune system,” “neural networks,” and “feature extraction” belong to this quadrant. Immunoinformatics combines immunology and bioinformatics to analyze immune system data, predict immune responses, and develop computational models for vaccine design and epitope prediction. The keywords “immune system,” “epitope,” and “immunoinformatics” are closely related, as epitopes are specific regions of antigens critical for vaccine development and immunotherapy. Neural networks show great potential for enhancing epitope prediction models and analyzing immune infiltration data. These keywords represent important interdisciplinary research areas between AI and immunology, which, despite their current limited impact on the broader field, have the potential to become core themes as AI becomes further integrated into these topics. (3) Lower Right—Basic Themes: Characterized by high centrality and low density, the themes in this quadrant are less researched but are gaining influence, with the potential to become central to future studies. Keywords in this quadrant include “random forest,” “classification,” “support vector machine,” “asthma,” “big data,” and “prediction.” ML algorithms like random forest and support vector machine (SVM) excel at classifying and predicting high-dimensional and heterogeneous data. As the volume of immunology and allergy data continues to grow, the ability to effectively analyze big data will be crucial to advancing research in this field. Future studies may focus on combining multiple classification algorithms to improve the accuracy and robustness of predictive models in immunology and allergy, particularly in diseases like asthma. Keywords such as “random forest,” “classification,” “support vector machine,” “asthma,” “big data,” and “prediction” represent emerging research themes in AI within the fields of immunology and allergy. As AI continues to evolve, these themes are likely to become central to the development of more accurate diagnostic tools, predictive models, and personalized treatment strategies. (4) Lower Left—Emerging or Declining Themes: This quadrant encompasses themes with low centrality and low density, typically representing areas that are either under-researched or declining in influence. “Artificial Immune Systems” is one such theme. AIS are computational systems inspired by the principles and processes of biological immune systems. They are advantageous in mimicking immune mechanisms such as pathogen recognition, memory, and adaptive learning. While AIS was the subject of significant research from 2007 to 2020, its prominence and importance in the field have notably declined in recent years.

**Figure 9:**
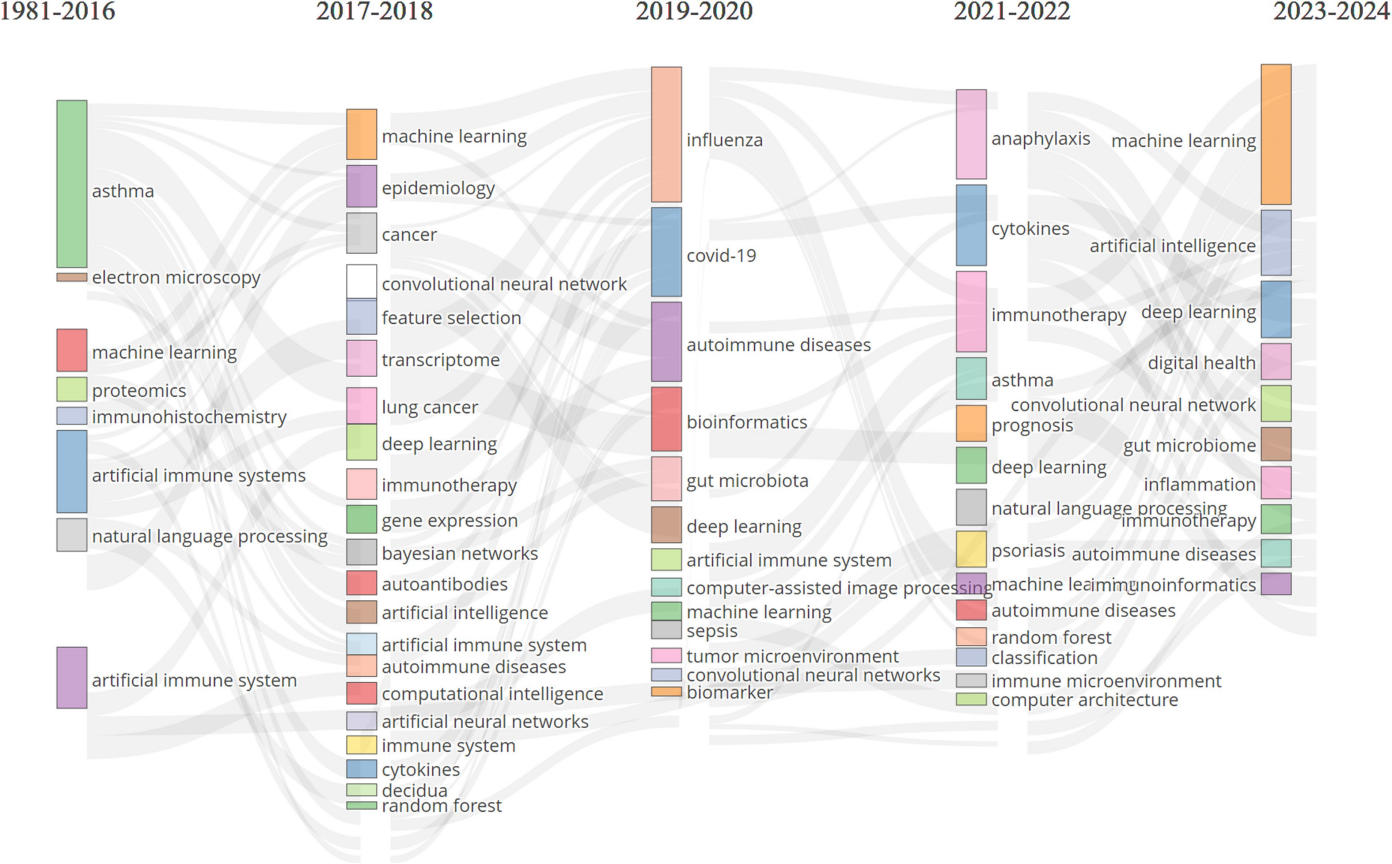
Thematic evolution on AI in allergy and immunology.

To further delineate the evolving themes within the field, we analyzed keywords that appeared more than eight times and divided the research into five phases, as illustrated in Figure 9: (1) Initial Phase (1986–2016): Research focused on topics such as “asthma,” “artificial immune systems,” “machine learning,” “proteomics,” “immunohistochemistry,” and “electron microscopy.” Early studies primarily targeted specific diseases, such as asthma, while developing foundational AI technologies like AIS and ML. Techniques such as proteomics, immunohistochemistry, and electron microscopy were crucial for understanding the molecular and cellular foundations of immune responses. The use of AIS reflected a growing interest in applying bio-inspired algorithms to simulate immune function. This period laid the groundwork for integrating AI with immunology and allergy research, particularly in disease modeling and molecular analysis. (2) 2017–2020: During this period, themes such as “machine learning,” “deep learning,” “epidemiology,” “cancer,” “transcriptome,” “gene expression,” “artificial intelligence,” and “immunotherapy” gained prominence. Broader topics such as epidemiology, cancer, and gene expression analysis related to immunology and allergy witnessed a marked shift in AI towards ML and DL. As research in these fields advanced, it generated vast amounts of high-dimensional, heterogeneous data, leading researchers to utilize more complex DL models for the analysis of extensive medical datasets. During this phase, immunotherapy became increasingly important, with growing research on optimizing cancer treatments and other immune-modulating therapies using AI. (3) 2019–2020: Key research hotspots during this phase included “COVID-19,” “influenza,” “autoimmune diseases,” “bioinformatics,” “gut microbiota,” “convolutional neural networks,” and “artificial immune systems.” COVID-19 became a dominant research focus, reflecting the global health crisis of this period. Researchers rapidly employed AI to track viral transmission, predict outcomes, and accelerate vaccine development. Convolutional neural networks (CNNs) and bioinformatics tools further enhanced the ability to analyze the vast amounts of genomic and clinical data associated with COVID-19. Interest in the gut microbiome and autoimmune diseases also grew during this time. The demand for simulating immune responses brought AIS research back into the spotlight. (4) 2021–2022: Themes such as “anaphylaxis,” “cytokines,” “asthma,” “immunotherapy,” “psoriasis,” “prognosis,” “autoimmune diseases,” and “deep learning” captured the attention of researchers. The focus of research during this period shifted toward more specific immune-related diseases, such as anaphylaxis, asthma, and psoriasis. DL continued to play an important role in analyzing immune data, while cytokine analysis became crucial for understanding immune responses, especially in the aftermath of COVID-19, as global health challenges persisted. During this phase, researchers increasingly employed AI to predict patient outcomes and guide treatment strategies. (5) 2023–2024: More recent themes, such as “machine learning,” “artificial intelligence,” “deep learning,” “digital health,” “gut microbiome,” “inflammation,” and “immunoinformatics,” have garnered greater attention. AI technologies like ML and DL continue to be central to immunology and allergy research. The emergence of new focus areas, such as digital health, highlights the integration of AI with healthcare delivery systems. Immunoinformatics, which combines immunology with bioinformatics, is gaining traction, reflecting the growing need for advanced computational tools to analyze immune data. Overall, the evolution of AI-related keywords in immunology and allergy research indicates that the field is increasingly reliant on advanced computational tools to address complex biological and medical problems. Clinically, attention is shifting toward AI-driven healthcare systems, with AI poised to become a key driver of research in immune mechanisms, personalized medicine, and digital health innovations.

### 3.6 Current research

Currently, the application of AI in immunology and allergy is focused on three primary directions: clinical research, basic research, and the development of drugs and immunotherapies. The rapid emergence of large-scale, high-dimensional, heterogeneous, and complex data—including imaging, speech, text, and omics data—has significantly increased the complexity of research and clinical diagnostics. Advances in deep CNNs and artificial neural networks (ANNs) have revolutionized image recognition technology, driving progress in histopathological immunology research and enhancing the ability to detect disease biomarkers and assist in diagnostics (91–94). Recurrent neural networks (RNNs), long short-term memory networks (LSTMs), and natural language processing (NLP) have dramatically improved the analysis of medical speech and text sequences. When combined with electronic health records (EHRs), these technologies further enhance early disease identification and management (95–99). Random forest algorithms and support vector machines (SVMs) have strengthened the ability to predict disease risk and progression based on patient histories, environmental factors, and genetic data (100–105). Meanwhile, in patient management and prognosis, applications such as chatbots (106, 107), and wearable devices (108, 109) have greatly facilitated self-management, improving patients’ quality of life.

Overall, AI and biological neuroscience have fostered a mutually reinforcing cycle of inspiration. The biological structure and function of the human brain have inspired the development of neural network models in AI (110, 111). These models, in turn, have been used to model and understand brain function and have further contributed to the prediction of factors, outcomes, and drug development for various immune diseases (112, 113). Metacognitive computing systems that simulate human thought processes provide insights into the development of more adaptive AI systems (114, 115). AI, particularly ML and DL, shows enormous promise in medical data analysis, aiding clinical decision-making, predicting diseases, improving patient care and experience, increasing healthcare system efficiency, reducing medical costs, and advancing medical education (2, 3, 116, 117). Advances in algorithms and training techniques, such as transfer learning, backpropagation, and dropout techniques, along with the expansion of computational resources (e.g., GPUs and cloud computing) and the development of genomic sequencing technologies and EHR systems, have driven the application of AI in allergy and immunology. As AI continues to evolve, it is increasingly capable of handling large-scale medical data, while advances in allergy and immunology provide more reliable datasets for AI applications. This mutual reinforcement has made AI an increasingly critical tool in allergy and immunology.

Additionally, AI-driven drug development has transformed immunology by accelerating the discovery of novel immunotherapies, optimizing vaccine design, and improving treatment personalization (112, 118, 119). ML and DL models are increasingly used to predict neoantigen-based immunotherapies, facilitating the development of personalized cancer vaccines by identifying highly immunogenic tumor epitopes (119). AI-enhanced bioinformatics has also contributed to the design of hypoallergenic derivatives, where ML models analyze allergen structures, modify epitopes to reduce immunogenicity, and ensure therapeutic efficacy, as seen in recent computational approaches to food allergy treatments (120, 121). Moreover, AI in epitope prediction enables precise identification of T-cell and B-cell epitopes, utilizing CNNs and generative adversarial networks (GANs) to model antigen–antibody interactions and predict immune responses more accurately than traditional methods (122, 123). AI-driven molecular docking further enhances therapeutic development by rapidly screening potential drug candidates, predicting their binding affinities to immune targets, and optimizing molecular modifications to improve efficacy and reduce side effects (124). Beyond drug discovery, AI significantly impacts allergy and immunology by improving prediction and prevention, where AI models forecast allergic reactions based on genomic, environmental, and clinical data, enabling early intervention (105); optimizing hospital operations through predictive analytics for immunotherapy scheduling and automated triage systems (125); enhancing patient care with AI-powered virtual assistants, wearable biosensors, and personalized treatment planning (126); and advancing diagnostics via deep learning-assisted imaging, immunological biomarker discovery, and AI-enhanced allergen testing to increase diagnostic accuracy (127). As AI continues to evolve, integrating these innovations into clinical practice will revolutionize immunological research, making therapies more effective, accessible, and personalized.

### 3.7 Current challenges

The development and application of AI in the fields of immunology and allergy are far from straightforward and face numerous challenges that must be addressed for AI to achieve its full potential. One of the fundamental issues is the quality of the data on which AI models rely. The datasets trained on these models inherently determine their performance. Given the intricate interplay of genetic, environmental, and lifestyle factors, collecting and standardizing large volumes of high-quality data to model human immune responses is no small feat. Poor data quality can introduce bias into AI-generated outputs, while insufficient data volumes can render AI models unreliable (31). The complexity of data collection, storage, and annotation further compounds the difficulty, significantly increasing the time and resource burden on researchers. Inconsistent data sets not only hamper the training of robust AI models but also limit their generalizability across different populations and clinical settings. Moreover, much of the training data currently comes from countries such as the USA, China, Australia, Japan, India, and European nations. This geographical concentration can lead to biased AI models, which may exhibit reduced effectiveness or even provide inaccurate results when applied to populations in other regions. The global applicability of AI models remains a significant challenge, particularly when attempting to deploy these models in resource-limited settings.

Another critical issue in this field is the lack of publicly accessible, high-quality datasets. Due to concerns over patient privacy, a lack of data-sharing infrastructure, and varying policies, the availability of comprehensive datasets remains limited, which in turn restricts the advancement of AI technologies in allergy and immunology. The future success of AI in these domains depends heavily on the creation of secure, shared data environments where researchers can collaborate and build more generalizable models.

Furthermore, the rapid advancement of AI technology has led to the development of increasingly complex models, which often function as “black boxes” (128). This lack of interpretability is a significant barrier to the clinical application of AI, as clinicians may struggle to understand the logic behind AI-driven recommendations (129, 130). When the reasoning process of an AI system is opaque, it can erode trust among healthcare providers and patients alike, making it difficult to integrate AI into routine clinical workflows. This raises a critical question: How should medical education systems evolve to prepare healthcare professionals to work effectively with AI tools? Integrating AI education and training into medical curricula will be essential to bridging this gap and ensuring that AI becomes a trusted component of clinical practice.

From an ethical perspective, the integration of AI in medical research and practice brings new challenges. The use of AI to analyze large-scale data raises concerns about patient privacy and the potential exposure of sensitive health information. The possibility of training AI models on non-representative datasets, driven by profit motives, could result in biased and inequitable outcomes. These ethical concerns necessitate the development of robust regulatory frameworks that keep pace with the rapid evolution of AI technologies. Ensuring that AI applications in immunology and allergy are ethically sound, transparent, and accountable will be critical to their long-term success.

### 3.8 Future prospects

Given the advances in immunology, allergology, data science, and computational resources, coupled with the widespread success of AI in radiology, oncology, and dermatology, the future of AI in allergy and immunology holds the promise of transformative growth. These pivotal experiences provide a roadmap for AI’s application and development in the field of allergy and immunology, particularly in areas such as improving diagnostic accuracy of allergic conditions through image recognition, enhancing personalized treatment plans using predictive models, accelerating the discovery of allergy therapies through AI-driven drug development, analyzing patterns of allergic reactions, and creating risk prediction tools to foresee severe allergic responses or asthma exacerbations.

The success of AI in these domains has been largely driven by collaboration among data scientists, clinicians, and biostatisticians. Similarly, advancing AI in allergy and immunology will depend on close interdisciplinary collaboration, offering diverse perspectives that drive rapid progress. This expansion will strengthen collaborative research efforts and propel the development and refinement of diagnostic tools, treatment strategies, and personalized healthcare systems.

However, AI’s application in radiology, oncology, and dermatology faces significant challenges, such as data collection, model transparency, generalizability of conclusions, and ethical and regulatory concerns. These obstacles provide valuable lessons for AI’s future development in allergy and immunology.

Considering the current state of AI research and development in allergy and immunology, as well as successes and challenges in other medical fields, we anticipate four core areas where AI will impact allergy and immunology:

1. Data serves as the foundation for the success of AI. Resolving the current limitations of training datasets will be crucial in the coming years. One of the primary challenges is the quality and quantity of training data. AI models require vast amounts of high-quality data to perform effectively, and one promising solution is the use of synthetic data and data augmentation. These techniques can boost the amount of usable data, improving model robustness and accuracy. Additionally, advancements in transfer learning have enhanced the precision of training on small datasets, enabling researchers to maximize the utility of limited data. Furthermore, innovations in federated learning and swarm learning offer effective solutions to privacy and security concerns. These approaches enable the training of models on large, distributed datasets, maintain data localization, and eliminate the need to pool sensitive patient data. This ensures a higher degree of security and privacy. Simultaneously, the advancement of these novel technologies offers a technological remedy for collaboration between countries abundant in resources and countries lacking resources in this domain. In the fields of immunology and allergy, these AI techniques will be invaluable in overcoming current challenges related to data quantity, quality, and privacy. As research progresses, these AI models will become increasingly robust, accurate, and applicable to a broad range of patient populations.
2. AI will play a pivotal role in driving personalized medicine. It will further enhance disease prediction, tailor treatments to individual patients, and optimize therapeutic outcomes. Using AI with multi-omics data, which includes genomics, proteomics, transcriptomics, and other molecular data, will help find new biomarkers and molecular signatures linked to immune responses. This will make it possible for more accurate diagnoses and treatments. These predictive models will assess an individual’s risk of developing allergic or immune-related conditions by considering genetic predispositions, lifestyle factors, and environmental exposures. This will enable the implementation of personalized preventive strategies tailored to the specific needs of each patient.
3. AI will enable researchers to more effectively identify promising drug candidates, continuing to accelerate drug development in immunology and allergy. Researchers will increasingly use ML and DL algorithms to screen chemical libraries, predict molecular interactions, and optimize drug formulations. AI will also play a critical role in the development of new biologics, including monoclonal antibodies and vaccines, by simulating immune responses and predicting patient outcomes. Additionally, AI’s ability to analyze large datasets will be essential for repurposing existing drugs for new therapeutic uses. By identifying similarities between disease pathways and drug targets, AI can expedite the discovery of new applications for existing medications, reducing the time and cost associated with traditional drug development. Furthermore, AI can play a pivotal role in predicting and mitigating adverse drug reactions by analyzing patient data to identify those most likely to experience specific side effects.
4. The future success of AI in immunology and allergy will depend on the creation of robust ethical and regulatory frameworks to address issues of data privacy, model transparency, and fairness. The reliance of AI on large-scale data prompts inquiries into the collection, storage, and utilization of patient information. It will be crucial to develop and deploy AI systems in a way that protects patient privacy and prevents the misuse of sensitive health data. Moreover, as AI models become increasingly complex, the need for transparency and interpretability will grow. Clinicians and patients must trust AI-driven recommendations, which means that AI systems need to clearly and transparently explain their decision-making processes. Addressing the “black box” problem will be essential for the broader adoption of AI in clinical practice. Another key ethical issue is ensuring that AI models do not exacerbate existing health disparities. AI systems developed from non-representative datasets may produce biased outcomes, disproportionately affecting marginalized populations. To avoid these biases and ensure equitable healthcare outcomes, it is crucial to train AI models on diverse datasets that represent the full spectrum of patient populations. Consequently, there is a worldwide demand for greater allocation of resources in this field in economically disadvantaged developed countries, as well as for enhanced collaboration between these countries and resource-poor developed countries. The goal is to increase the proportion of data sets in this field within these countries, as well as maximize data representation. Finally, regulatory bodies will need to keep pace with the rapid evolution of AI technologies. Developing standardized guidelines for the validation, testing, and deployment of AI models in clinical settings will be essential to ensuring their safety and effectiveness. Given the global nature of healthcare and the cross-border application of AI systems, international collaboration on regulatory standards will be particularly important.

Moreover, in the face of growing AI, a number of other questions arise, such as whether a reduction in interpersonal interactions will increase the frequency of psychological problems (131). Over-reliance on AI may weaken the patient-physician relationship, leading to a decline in empathy and patient satisfaction. Personal interactions between patients and healthcare providers are critical to effective care, and AI should augment, not replace, this dynamic. At the same time, relying on AI for decision-making may weaken the critical thinking skills of students and clinicians (132, 133), and may lead to diagnostic or treatment errors if the AI system malfunctions or provides incorrect advice. This set of issues deserves to be explored in depth. Overall, addressing these risks requires adequate research, developing ethical guidelines, implementing unbiased training datasets, ensuring transparency in the AI decision-making process, and establishing robust data protection regulations. By proactively addressing these challenges, the healthcare industry can fully utilize the potential of AI while safeguarding patient rights and maintaining the integrity of clinical practice.

## 4 Limitations

This study solely relied on data from the WoSCC due to the limitations of current bibliometric tools, potentially missing articles indexed in other databases like Scopus and PubMed that WoSCC does not cover. Furthermore, impact analysis focused primarily on citation counts, which may underestimate the influence of recently published articles. In future research, we plan to incorporate information from other databases and improve research methodologies to address these limitations.

## 5 Conclusion

Our findings indicate that 21,552 authors have published 3,883 related papers across 1,247 journals, with the USA and China leading in output and impact in the field of AI in allergy and immunology, and trends suggest that their lead is expanding. Overall, the field of allergy and immunology has widely applied AI, particularly ML and DL, for drug prediction and development, classification and prediction of allergic and immunological diseases, diagnostic assistance, immunological and genomic modeling, decision support, digitalization, smart integration of healthcare systems, and medical education. The field is evolving to integrate AI technologies with specific clinical applications for personalized medicine, offering significant future application prospects.

However, development in this field is not without challenges, including technical, ethical, and regulatory hurdles. Addressing these challenges requires the collective effort of policymakers, educators, researchers, healthcare workers, and funding agencies. Creating a more inclusive and equitable AI research environment in allergy and immunology, ensuring that AI positively impacts global health, advancing technology, and improving global health and well-being remain critical.

## Data Availability

The original contributions presented in the study are included in the article/supplementary material, further inquiries can be directed to the corresponding author.

## References

[1] Kaplan A Cao H Fitzgerald JM Iannotti N Yang E Kocks JW . Artificial intelligence/machine learning in respiratory medicine and potential role in asthma and Copd diagnosis. J Allergy Clin Immunol Pract. (2021) 9:2255–61. doi: 10.1016/j.jaip.2021.02.014, PMID: 33618053

[2] Khoury P Srinivasan R Kakumanu S Ochoa S Keswani A Sparks R . A framework for augmented intelligence in allergy and immunology practice and research—a work group report of the AAAAI health informatics, technology, and education committee. J Allergy Clin Immunol Pract. (2022) 10:1178–88. doi: 10.1016/j.jaip.2022.01.047, PMID: 35300959 PMC9205719

[3] Topol EJ. High-performance medicine: the convergence of human and artificial intelligence. Nat Med. (2019) 25:44–56. doi: 10.1038/s41591-018-0300-7, PMID: 30617339

[4] Janiesch C Zschech P Heinrich K. Machine learning and deep learning. Electron Mark. (2021) 31:685–95. doi: 10.1007/s12525-021-00475-2, PMID: 40151768

[5] Lecun Y Bengio Y Hinton G. Deep learning. Nature. (2015) 521:436–44. doi: 10.1038/nature14539, PMID: 26017442

[6] Krizhevsky A Sutskever I Hinton GE. Imagenet classification with deep convolutional neural networks. Adv Neural Inf Proces Syst. (2012) 25

[7] Sainath TN Kingsbury B Saon G Soltau H Mohamed A-R Dahl G . Deep convolutional neural networks for large-scale speech tasks. Neural Netw. (2015) 64:39–48. doi: 10.1016/j.neunet.2014.08.005, PMID: 25439765

[8] Ma J Sheridan RP Liaw A Dahl GE Svetnik V. Deep neural nets as a method for quantitative structure–activity relationships. J Chem Inf Model. (2015) 55:263–74. doi: 10.1021/ci500747n, PMID: 25635324

[9] Xiong HY Alipanahi B Lee LJ Bretschneider H Merico D Yuen RK . The human splicing code reveals new insights into the genetic determinants of disease. Science. (2015) 347:1254806. doi: 10.1126/science.1254806 25525159 PMC4362528

[10] Obermeyer Z Emanuel EJ. Predicting the future—big data, machine learning, and clinical medicine. N Engl J Med. (2016) 375:1216. doi: 10.1056/NEJMp1606181, PMID: 27682033 PMC5070532

[11] Pickett G Motazedi T Kutac C Cahill G Cunnigham-Rundles C Fuleihan RL . Infection phenotypes among patients with primary antibody deficiency mined from a us patient registry. J Clin Immunol. (2021) 41:374–81. doi: 10.1007/s10875-020-00916-1, PMID: 33205244

[12] Morley J Machado CC Burr C Cowls J Joshi I Taddeo M . The ethics of Ai in health care: a mapping review. Soc Sci Med. (2020) 260:113172. doi: 10.1016/j.socscimed.2020.113172, PMID: 32702587

[13] Ietswaart R Arat S Chen AX Farahmand S Kim B Dumouchel W . Machine learning guided association of adverse drug reactions with in vitro target-based pharmacology. EBioMedicine. (2020) 57:102837. doi: 10.1016/j.ebiom.2020.102837, PMID: 32565027 PMC7379147

[14] Vamathevan J Clark D Czodrowski P Dunham I Ferran E Lee G . Applications of machine learning in drug discovery and development. Nat Rev Drug Discov. (2019) 18:463–77. doi: 10.1038/s41573-019-0024-5, PMID: 30976107 PMC6552674

[15] Goodwin S Mcpherson JD Mccombie WR. Coming of age: ten years of next-generation sequencing technologies. Nat Rev Genet. (2016) 17:333–51. doi: 10.1038/nrg.2016.49, PMID: 27184599 PMC10373632

[16] Dymek C Kim B Melton GB Payne TH Singh H Hsiao C-J. Building the evidence-base to reduce electronic health record–related clinician burden. J Am Med Inform Assoc. (2021) 28:1057–61. doi: 10.1093/jamia/ocaa238, PMID: 33340326 PMC8068419

[17] Xiao N Huang X Zang W Kiselev S Bolkov MA Tuzankina IA . Health-related quality of life in patients with inborn errors of immunity: a bibliometric analysis. Front Immunol. (2024) 15:1371124. doi: 10.3389/fimmu.2024.1371124, PMID: 38515759 PMC10954858

[18] Zang W Chen H Yan J Li D Xiao N Zheng X . Research trends and hotspots of exercise for people with sarcopenic: a bibliometric analysis. Medicine. (2023) 102:e35148. doi: 10.1097/MD.0000000000035148, PMID: 38115285 PMC10727540

[19] Xiao N Huang X Li B Sun L Kiselev S. Effects of digital environments on children’s cognitive function and mental health: a bibliometric study. Curr Psychol. (2024) 43:28038–52. doi: 10.1007/s12144-024-06476-6, PMID: 40151768

[20] Chen C. Searching for intellectual turning points: progressive knowledge domain visualization. Proc Natl Acad Sci. (2004) 101:5303–10. doi: 10.1073/pnas.0307513100, PMID: 14724295 PMC387312

[21] Haroske G Haroske C Haroske K. Image processing in pathology: X. Electron microscopic morphometric analysis of human lymphocyte subpopulations. Exp Pathol. (1981) 19:67–80. doi: 10.1016/S0232-1513(81)80037-0, PMID: 6974097

[22] Farmer JD Packard NH Perelson AS. The immune system, adaptation, and machine learning. Physica D. (1986) 22:187–204. doi: 10.1016/0167-2789(86)90240-X

[23] Oksel C Haider S Fontanella S Frainay C Custovic A. Classification of pediatric asthma: from phenotype discovery to clinical practice. Front Pediatr. (2018) 6:258. doi: 10.3389/fped.2018.00258, PMID: 30298124 PMC6160736

[24] Prosperi MC Marinho S Simpson A Custovic A Buchan IE. Predicting phenotypes of asthma and eczema with machine learning. BMC Med Genet. (2014) 7:1–10. doi: 10.1186/1755-8794-7-S1-S7 PMC4101570 25077568

[25] Prosperi MC Belgrave D Buchan I Simpson A Custovic A. Challenges in interpreting allergen microarrays in relation to clinical symptoms: a machine learning approach. Pediatr Allergy Immunol. (2014) 25:71–9. doi: 10.1111/pai.12139, PMID: 24131308 PMC4282342

[26] Deliu M Fontanella S Haider S Sperrin M Geifman N Murray C . Longitudinal trajectories of severe wheeze exacerbations from infancy to school age and their association with early-life risk factors and late asthma outcomes. Clin Exp Allergy. (2020) 50:315–24. doi: 10.1111/cea.13553, PMID: 31876035 PMC7065181

[27] Howard R Fontanella S Simpson A Murray CS Custovic A Rattray M. Component-specific clusters for diagnosis and prediction of allergic airway diseases. Clin Exp Allergy. (2024) 54:339–49. doi: 10.1111/cea.14468, PMID: 38475973

[28] Simpson A Tan VY Winn J Svensen M Bishop CM Heckerman DE . Beyond atopy: multiple patterns of sensitization in relation to asthma in a birth cohort study. Am J Respir Crit Care Med. (2010) 181:1200–6. doi: 10.1164/rccm.200907-1101OC, PMID: 20167852

[29] Kothalawala DM Murray CS Simpson A Custovic A Tapper WJ Arshad SH . Development of childhood asthma prediction models using machine learning approaches. Clin Transl Allergy. (2021) 11:e12076. doi: 10.1002/clt2.12076 34841728 PMC9815427

[30] Fontanella S Cucco A Custovic A. Machine learning in asthma research: moving toward a more integrated approach. Expert Rev Respir Med. (2021) 15:609–21. doi: 10.1080/17476348.2021.1894133, PMID: 33618597

[31] Van Breugel M Fehrmann RS Bügel M Rezwan FI Holloway JW Nawijn MC . Current state and prospects of artificial intelligence in allergy. Allergy. (2023) 78:2623–43. doi: 10.1111/all.15849, PMID: 37584170

[32] Alvarez B Reynisson B Barra C Buus S Ternette N Connelley T . Nnalign_Ma; Mhc peptidome deconvolution for accurate Mhc binding motif characterization and improved T-cell epitope predictions. Mol Cell Proteomics. (2019) 18:2459–77. doi: 10.1074/mcp.TIR119.001658, PMID: 31578220 PMC6885703

[33] Nielsen M Lund O Buus S Lundegaard C. Mhc class ii epitope predictive algorithms. Immunology. (2010) 130:319–28. doi: 10.1111/j.1365-2567.2010.03268.x, PMID: 20408898 PMC2913211

[34] Paul S Croft NP Purcell AW Tscharke DC Sette A Nielsen M . Benchmarking predictions of Mhc class I restricted T cell epitopes in a comprehensively studied model system. PLoS Comput Biol. (2020) 16:e1007757. doi: 10.1371/journal.pcbi.1007757, PMID: 32453790 PMC7274474

[35] Nielsen M Ternette N Barra C. The interdependence of machine learning and Lc-Ms approaches for an unbiased understanding of the cellular immunopeptidome. Expert Rev Proteomics. (2022) 19:77–88. doi: 10.1080/14789450.2022.2064278, PMID: 35390265

[36] Barra C Ackaert C Reynisson B Schockaert J Jessen LE Watson M . Immunopeptidomic data integration to artificial neural networks enhances protein-drug immunogenicity prediction. Front Immunol. (2020) 11:1304. doi: 10.3389/fimmu.2020.01304 32655572 PMC7325480

[37] Lazic N Roberts G Custovic A Belgrave D Bishop C Winn J . Multiple atopy phenotypes and their associations with asthma: similar findings from two birth cohorts. Allergy. (2013) 68:764–70. doi: 10.1111/all.12134, PMID: 23621120

[38] Prosperi MC Sahiner UM Belgrave D Sackesen C Buchan IE Simpson A . Challenges in identifying asthma subgroups using unsupervised statistical learning techniques. Am J Respir Crit Care Med. (2013) 188:1303–12. doi: 10.1164/rccm.201304-0694OC, PMID: 24180417 PMC3919072

[39] Lin L. Curtin J. A. Regis E. Hirsman A. Howard R. Tutino M. . (2022). A systems immunology approach to investigate cytokine responses to viruses and bacteria and their association with disease. Sci Rep, 12,:13463, doi: 10.1038/s41598-022-16509-4, PMID: , PMCID: . 35931775 PMC9356009

[40] Wu J Prosperi MC Simpson A Hollams EM Sly PD Custovic A . Relationship between cytokine expression patterns and clinical outcomes: two population-based birth cohorts. Clin Exp Allergy. (2015) 45:1801–11. doi: 10.1111/cea.12579, PMID: 26061524 PMC4950290

[41] Luo G Johnson MD Nkoy FL He S Stone BL. Automatically explaining machine learning prediction results on asthma hospital visits in patients with asthma: secondary analysis. JMIR Med Inform. (2020) 8:e21965. doi: 10.2196/21965 33382379 PMC7808890

[42] Luo G Stone BL Fassl B Maloney CG Gesteland PH Yerram SR . Predicting asthma control deterioration in children. BMC Med Inform Decis Mak. (2015) 15:1–8. doi: 10.1186/s12911-015-0208-9 26467091 PMC4607145

[43] Luo G. A roadmap for automating lineage tracing to aid automatically explaining machine learning predictions for clinical decision support. JMIR Med Inform. (2021) 9:e27778. doi: 10.2196/27778, PMID: 34042600 PMC8193496

[44] Tong Y Messinger AI Wilcox AB Mooney SD Davidson GH Suri P . Forecasting future asthma hospital encounters of patients with asthma in an academic health care system: predictive model development and secondary analysis study. J Med Internet Res. (2021) 23:e22796. doi: 10.2196/22796, PMID: 33861206 PMC8087967

[45] Kang M Ko E Mersha TB. A roadmap for multi-omics data integration using deep learning. Brief Bioinform. (2022) 23:bbab454. doi: 10.1093/bib/bbab454 34791014 PMC8769688

[46] Ko E Kim Y Shokoohi F Mersha TB Kang M. Spin: sex-specific and pathway-based interpretable neural network for sexual dimorphism analysis. Brief Bioinform. (2024) 25:bbae239. doi: 10.1093/bib/bbae239 38807262 PMC11133003

[47] Proper SP Azouz NP Mersha TB. Achieving precision medicine in allergic disease: progress and challenges. Front Immunol. (2021) 12:720746. doi: 10.3389/fimmu.2021.720746, PMID: 34484229 PMC8416451

[48] Dessie EY Gautam Y Ding L Altaye M Beyene J Mersha TB. Development and validation of asthma risk prediction models using co-expression gene modules and machine learning methods. Sci Rep. (2023) 13:11279. doi: 10.1038/s41598-023-35866-2 37438356 PMC10338542

[49] Banerji A Lai KH Li Y Saff RR Camargo CA Jr Blumenthal KG . Natural language processing combined with Icd-9-cm codes as a novel method to study the epidemiology of allergic drug reactions. J Allergy Clin Immunol Pract. (2020) 8:1032–8. e1. doi: 10.1016/j.jaip.2019.12.007, PMID: 31857264 PMC7064405

[50] Alvarez-Arango S Yerneni S Tang O Zhou L Mancini CM Blackley SV . Vancomycin hypersensitivity reactions documented in electronic health records. J Allergy Clin Immunol Pract. (2021) 9:906–12. doi: 10.1016/j.jaip.2020.09.027, PMID: 33011300 PMC7870516

[51] Wang L Blackley SV Blumenthal KG Yerneni S Goss FR Lo Y-C . A dynamic reaction picklist for improving allergy reaction documentation in the electronic health record. J Am Med Inform Assoc. (2020) 27:917–23. doi: 10.1093/jamia/ocaa042, PMID: 32417930 PMC7309236

[52] Yang J Wang L Phadke NA Wickner PG Mancini CM Blumenthal KG . Development and validation of a deep learning model for detection of allergic reactions using safety event reports across hospitals. JAMA Netw Open. (2020) 3:e2022836–6. doi: 10.1001/jamanetworkopen.2020.22836, PMID: 33196805 PMC7670315

[53] Hurvitz N Azmanov H Kesler A Ilan Y. Establishing a second-generation artificial intelligence-based system for improving diagnosis, treatment, and monitoring of patients with rare diseases. Eur J Hum Genet. (2021) 29:1485–90. doi: 10.1038/s41431-021-00928-4, PMID: 34276056 PMC8484657

[54] Azmanov H Bayatra A Ilan Y. Digital analgesic comprising a second-generation digital health system: increasing effectiveness by optimizing the dosing and minimizing side effects. J Pain Res. (2022) 15:1051–60. doi: 10.2147/JPR.S356319, PMID: 35444460 PMC9013915

[55] Kolben Y Azmanov H Gelman R Dror D Ilan Y. Using chronobiology-based second-generation artificial intelligence digital system for overcoming antimicrobial drug resistance in chronic infections. Ann Med. (2023) 55:311–8. doi: 10.1080/07853890.2022.2163053, PMID: 36594558 PMC9815249

[56] Ilan Y. Improving global healthcare and reducing costs using second-generation artificial intelligence-based digital pills: a market disruptor. Int J Environ Res Public Health. (2021) 18:811. doi: 10.3390/ijerph18020811, PMID: 33477865 PMC7832873

[57] Andreatta M Jurtz VI Kaever T Sette A Peters B Nielsen M. Machine learning reveals a non-canonical mode of peptide binding to Mhc class ii molecules. Immunology. (2017) 152:255–64. doi: 10.1111/imm.12763, PMID: 28542831 PMC5588850

[58] Jurtz V Paul S Andreatta M Marcatili P Peters B Nielsen M. Netmhcpan-4.0: improved peptide–Mhc class I interaction predictions integrating eluted ligand and peptide binding affinity data. J Immunol. (2017) 199:3360–8. doi: 10.4049/jimmunol.1700893, PMID: 28978689 PMC5679736

[59] Reynisson B Barra C Kaabinejadian S Hildebrand WH Peters B Nielsen M. Improved prediction of Mhc ii antigen presentation through integration and motif deconvolution of mass spectrometry Mhc eluted ligand data. J Proteome Res. (2020) 19:2304–15. doi: 10.1021/acs.jproteome.9b00874, PMID: 32308001

[60] Clifford JN Høie MH Deleuran S Peters B Nielsen M Marcatili P. BepiPred-3.0: improved B-cell epitope prediction using protein language models. Protein Sci. (2022) 31:e4497. doi: 10.1002/pro.4497, PMID: 36366745 PMC9679979

[61] Peters B Nielsen M Sette A. T cell epitope predictions. Annu Rev Immunol. (2020) 38:123–45. doi: 10.1146/annurev-immunol-082119-124838, PMID: 32045313 PMC10878398

[62] Kumar N Tripathi S Sharma N Patiyal S Devi NL Raghava GP. A method for predicting linear and conformational B-cell epitopes in an antigen from its primary sequence. Comput Biol Med. (2024) 170:108083. doi: 10.1016/j.compbiomed.2024.108083, PMID: 38295479

[63] Kaur D Arora C Raghava GP. A hybrid model for predicting pattern recognition receptors using evolutionary information. Front Immunol. (2020) 11:512902. doi: 10.3389/fimmu.2020.00071, PMID: 32082326 PMC7002473

[64] Kaur D Patiyal S Arora C Singh R Lodhi G Raghava GP. In-silico tool for predicting, scanning, and designing defensins. Front Immunol. (2021) 12:780610. doi: 10.3389/fimmu.2021.780610, PMID: 34880873 PMC8645896

[65] Himes BE Wu AC Duan QL Klanderman B Litonjua AA Tantisira K . Predicting response to short-acting bronchodilator medication using Bayesian networks. Pharmacogenomics. (2009) 10:1393–412. doi: 10.2217/pgs.09.93, PMID: 19761364 PMC2804237

[66] Chu J-H Weiss ST Carey VJ Raby BA. A graphical model approach for inferring large-scale networks integrating gene expression and genetic polymorphism. BMC Syst Biol. (2009) 3:1–9. doi: 10.1186/1752-0509-3-55 19473523 PMC2694152

[67] Park H-W Weiss ST. Understanding the molecular mechanisms of asthma through transcriptomics. Allergy, Asthma Immunol Res. (2020) 12:399. doi: 10.4168/aair.2020.12.3.399, PMID: 32141255 PMC7061151

[68] Kelly RS Mcgeachie MJ Lee-Sarwar KA Kachroo P Chu SH Virkud YV . Partial least squares discriminant analysis and Bayesian networks for metabolomic prediction of childhood asthma. Meta. (2018) 8:68. doi: 10.3390/metabo8040068, PMID: 30360514 PMC6316795

[69] Peer K Adams WG Legler A Sandel M Levy JI Boynton-Jarrett R . Developing and evaluating a pediatric asthma severity computable phenotype derived from electronic health records. J Allergy Clin Immunol. (2021) 147:2162–70. doi: 10.1016/j.jaci.2020.11.045, PMID: 33338540 PMC8328264

[70] Zhang Y-H Li Z Zeng T Lu W Huang T Cai Y-D. Identifying the immunological gene signatures of immune cell subtypes. Biomed Res Int. (2021) 2021:6639698. doi: 10.1155/2021/6639698, PMID: 39959028

[71] Chen L Mei Z Guo W Ding S Huang T Cai Y-D. Recognition of immune cell markers of Covid-19 severity with machine learning methods. Biomed Res Int. (2022) 2022:6089242. doi: 10.1155/2022/6089242, PMID: 35528178 PMC9073549

[72] Li H Huang F Liao H Li Z Feng K Huang T . Identification of Covid-19-specific immune markers using a machine learning method. Front Mol Biosci. (2022) 9:952626. doi: 10.3389/fmolb.2022.952626, PMID: 35928229 PMC9344575

[73] Polat K Güneş S. Medical decision support system based on artificial immune recognition immune system (Airs), fuzzy weighted pre-processing and feature selection. Expert Syst Appl. (2007) 33:484–90. doi: 10.1016/j.eswa.2006.05.013

[74] Polat K Güneş S. Computer aided medical diagnosis system based on principal component analysis and artificial immune recognition system classifier algorithm. Expert Syst Appl. (2008) 34:773–9. doi: 10.1016/j.eswa.2006.10.011

[75] Bidoki NH Zera KA Nassar H Drag LL Mlynash M Osborn E . Machine learning models of plasma proteomic data predict mood in chronic stroke and tie it to aberrant peripheral immune responses. Brain Behav Immun. (2023) 114:144–53. doi: 10.1016/j.bbi.2023.08.002, PMID: 37557961 PMC10792657

[76] Culos A Tsai AS Stanley N Becker M Ghaemi MS Mcilwain DR . Integration of mechanistic immunological knowledge into a machine learning pipeline improves predictions. Nat Mach Intell. (2020) 2:619–28. doi: 10.1038/s42256-020-00232-8, PMID: 33294774 PMC7720904

[77] Zhang GL Lin HH Keskin DB Reinherz EL Brusic V. Dana-Farber repository for machine learning in immunology. J Immunol Methods. (2011) 374:18–25. doi: 10.1016/j.jim.2011.07.007, PMID: 21782820 PMC3249226

[78] Zhang GL Sun J Chitkushev L Brusic V. Big data analytics in immunology: a knowledge-based approach. Biomed Res Int. (2014) 2014:437987. doi: 10.1155/2014/645056, PMID: 25045677 PMC4090507

[79] Von Ungern-Sternberg S. Bradford's law in the context of information provision. Scientometrics. (2000) 49:161–86. doi: 10.1023/A:1005669410627

[80] Finkelstein J Jeong IC. Machine learning approaches to personalize early prediction of asthma exacerbations. Ann N Y Acad Sci. (2017) 1387:153–65. doi: 10.1111/nyas.13218, PMID: 27627195 PMC5266630

[81] Hoof I Peters B Sidney J Pedersen LE Sette A Lund O . Netmhcpan, a method for Mhc class I binding prediction beyond humans. Immunogenetics. (2009) 61:1–13. doi: 10.1007/s00251-008-0341-z, PMID: 19002680 PMC3319061

[82] Jespersen MC Peters B Nielsen M Marcatili P. BepiPred-2.0: improving sequence-based B-cell epitope prediction using conformational epitopes. Nucleic Acids Res. (2017) 45:W24–9. doi: 10.1093/nar/gkx346, PMID: 28472356 PMC5570230

[83] Dimitrov I Bangov I Flower DR Doytchinova I. Allertop v. 2—a server for in silico prediction of allergens. J Mol Model. (2014) 20:1–6. doi: 10.1007/s00894-014-2278-5 24878803

[84] Howard R Rattray M Prosperi M Custovic A. Distinguishing asthma phenotypes using machine learning approaches. Curr Allergy Asthma Rep. (2015) 15:38. doi: 10.1007/s11882-015-0542-0 26143394 PMC4586004

[85] Luo G He S Stone BL Nkoy FL Johnson MD. Developing a model to predict hospital encounters for asthma in asthmatic patients: secondary analysis. JMIR Med Inform. (2020) 8:e16080. doi: 10.2196/16080 31961332 PMC7001050

[86] Sidhom J-W Larman HB Pardoll DM Baras AS. Deeptcr is a deep learning framework for revealing sequence concepts within T-cell repertoires. Nat Commun. (2021) 12:1605. doi: 10.1038/s41467-021-21879-w 33707415 PMC7952906

[87] Exarchos KP Beltsiou M Votti C-A Kostikas K. Artificial intelligence techniques in asthma: a systematic review and critical appraisal of the existing literature. Eur Respir J. (2020) 56:2000521. doi: 10.1183/13993003.00521-2020 32381498

[88] Wi C-I Sohn S Rolfes MC Seabright A Ryu E Voge G . Application of a natural language processing algorithm to asthma ascertainment. An automated chart review. Am J Respir Crit Care Med. (2017) 196:430–7. doi: 10.1164/rccm.201610-2006OC, PMID: 28375665 PMC5564673

[89] Timmis J Neal M Hunt J. An artificial immune system for data analysis. Biosystems. (2000) 55:143–50. doi: 10.1016/S0303-2647(99)00092-1, PMID: 10745118

[90] Dasgupta D Yu S Nino F. Recent advances in artificial immune systems: models and applications. Appl Soft Comput. (2011) 11:1574–87. doi: 10.1016/j.asoc.2010.08.024, PMID: 40151496

[91] Junayed M. S. Sakib A. N. M. Anjum N. Islam M. B. Jeny A. A. EczemaNet: a deep Cnn-based eczema diseases classification. In 2020 IEEE 4th International Conference on Image Processing, Applications and Systems (IPAS), (2020). IEEE, 174–179.

[92] Khan A Sohail A Zahoora U Qureshi AS. A survey of the recent architectures of deep convolutional neural networks. Artif Intell Rev. (2020) 53:5455–516. doi: 10.1007/s10462-020-09825-6

[93] Qin Y Wang J Han Y Lu L. Deep learning algorithms-based Ct images in glucocorticoid therapy in Asthma children with small airway obstruction. J Healthcare Eng. (2021) 2021:5317403. doi: 10.1155/2021/5317403, PMID: 34721824 PMC8553479

[94] Rasheed A Umar AI Shirazi SH Khan Z Nawaz S Shahzad M. Automatic eczema classification in clinical images based on hybrid deep neural network. Comput Biol Med. (2022) 147:105807. doi: 10.1016/j.compbiomed.2022.105807, PMID: 35809409

[95] Dreisbach C Koleck TA Bourne PE Bakken S. A systematic review of natural language processing and text mining of symptoms from electronic patient-authored text data. Int J Med Inform. (2019) 125:37–46. doi: 10.1016/j.ijmedinf.2019.02.008, PMID: 30914179 PMC6438188

[96] Hossain E Rana R Higgins N Soar J Barua PD Pisani AR . Natural language processing in electronic health records in relation to healthcare decision-making: a systematic review. Comput Biol Med. (2023) 155:106649. doi: 10.1016/j.compbiomed.2023.106649, PMID: 36805219

[97] Seol HY Rolfes MC Chung W Sohn S Ryu E Park MA . Expert artificial intelligence-based natural language processing characterises childhood asthma. BMJ Open Respir Res. (2020) 7:e000524. doi: 10.1136/bmjresp-2019-000524, PMID: 33371009 PMC7011897

[98] Wi C-I Sohn S Ali M Krusemark E Ryu E Liu H . Natural language processing for asthma ascertainment in different practice settings. J Allergy Clin Immunol Pract. (2018) 6:126–31. doi: 10.1016/j.jaip.2017.04.041, PMID: 28634104 PMC5733699

[99] Wi CI Sohn S Ryu E Liu H Park MA Juhn YJ. Automated chart review for asthma ascertainment: an innovative approach for asthma care and research in the era of electronic medical record. J Allergy Clin Immunol. (2016) 137:Ab196. doi: 10.1016/j.jaci.2015.12.771

[100] Custovic A Simpson BM Murray CS Lowe L Woodcock A Asthma NM . The national asthma campaign Manchester asthma and allergy study. Pediatr Allergy Immunol. (2002) 13:32–7. doi: 10.1034/j.1399-3038.13.s.15.3.x, PMID: 12688622

[101] Engelhardt KR Gertz ME Keles S Schäffer AA Sigmund EC Glocker C . The extended clinical phenotype of 64 patients with dedicator of cytokinesis 8 deficiency. J Allergy Clin Immunol. (2015) 136:402–12. doi: 10.1016/j.jaci.2014.12.1945, PMID: 25724123 PMC4530066

[102] Fortino V Wisgrill L Werner P Suomela S Linder N Jalonen E . Machine-learning–driven biomarker discovery for the discrimination between allergic and irritant contact dermatitis. Proc Natl Acad Sci. (2020) 117:33474–85. doi: 10.1073/pnas.2009192117, PMID: 33318199 PMC7776829

[103] Kamphorst K Lopez-Rincon A Vlieger AM Garssen J Van’t Riet E Van Elburg RM. Predictive factors for allergy at 4–6 years of age based on machine learning: a pilot study. PharmaNutrition. (2023) 23:100326. doi: 10.1016/j.phanu.2022.100326

[104] Kavya R Christopher J Panda S Lazarus YB. Machine learning and Xai approaches for allergy diagnosis. Biomed Signal Process Control. (2021) 69:102681. doi: 10.1016/j.bspc.2021.102681

[105] Shamji MH Ollert M Adcock IM Bennett O Favaro A Sarama R . Eaaci guidelines on environmental science in allergic diseases and asthma–leveraging artificial intelligence and machine learning to develop a causality model in exposomics. Allergy. (2023) 78:1742–57. doi: 10.1111/all.15667, PMID: 36740916

[106] Chaix B Bibault J-E Pienkowski A Delamon G Guillemassé A Nectoux P . When chatbots meet patients: one-year prospective study of conversations between patients with breast cancer and a chatbot. JMIR Cancer. (2019) 5:e12856. doi: 10.2196/12856, PMID: 31045505 PMC6521209

[107] Kadariya D. Venkataramanan R. Yip H. Y. Kalra M. Thirunarayanan K. Sheth A. kBot: knowledge-enabled personalized chatbot for asthma self-management. In 2019 IEEE International Conference on Smart Computing (Smartcomp), (2019). IEEE, 138–143. 10.1109/smartcomp.2019.00043 PMC7432964 32832938

[108] Fakotakis DN Nousias S Arvanitis G Zacharaki EI Moustakas K. Ai sound recognition on asthma medication adherence: evaluation with the Rda benchmark suite. IEEE Access. (2023) 11:13810–29. doi: 10.1109/ACCESS.2023.3243547

[109] Shim JS Kim MH Lee SM Kim SH Kwon JW Song C . An artificial intelligence algorithm-based smartphone application for daily cough monitoring. Allergy. (2023):78. doi: 10.1111/all.15632 36588171

[110] Goertzel B Lian R Arel I De Garis H Chen S. A world survey of artificial brain projects, part ii: biologically inspired cognitive architectures. Neurocomputing. (2010) 74:30–49. doi: 10.1016/j.neucom.2010.08.012

[111] Stanley KO Clune J Lehman J Miikkulainen R. Designing neural networks through neuroevolution. Nat Machine Intell. (2019) 1:24–35. doi: 10.1038/s42256-018-0006-z

[112] Moingeon P. Artificial intelligence-driven drug development against autoimmune diseases. Trends Pharmacol Sci. (2023) 44:411–24. doi: 10.1016/j.tips.2023.04.005, PMID: 37268540

[113] Zhou J Theesfeld CL Yao K Chen KM Wong AK Troyanskaya OG. Deep learning sequence-based ab initio prediction of variant effects on expression and disease risk. Nat Genet. (2018) 50:1171–9. doi: 10.1038/s41588-018-0160-6, PMID: 30013180 PMC6094955

[114] Callaway F Jain YR Van Opheusden B Das P Iwama G Gul S . Leveraging artificial intelligence to improve people’s planning strategies. Proc Natl Acad Sci. (2022) 119:e2117432119. doi: 10.1073/pnas.2117432119, PMID: 35294284 PMC8944825

[115] Wang JX. Meta-learning in natural and artificial intelligence. Curr Opin Behav Sci. (2021) 38:90–5. doi: 10.1016/j.cobeha.2021.01.002

[116] Matheny M Israni ST Ahmed M Whicher D. Artificial intelligence in health care: The hope, the hype, the promise, the peril. Dc: National Academy of Medicine Washington (2023). 39146448

[117] Stafford IS Kellermann M Mossotto E Beattie RM Macarthur BD Ennis S. A systematic review of the applications of artificial intelligence and machine learning in autoimmune diseases. NPJ Digit Med. (2020) 3:30. doi: 10.1038/s41746-020-0229-3, PMID: 32195365 PMC7062883

[118] Kumar A Dixit S Srinivasan K Vincent PDR. Personalized cancer vaccine design using Ai-powered technologies. Front Immunol. (2024) 15:1357217. doi: 10.3389/fimmu.2024.1357217, PMID: 39582860 PMC11581883

[119] Li Y Wu X Fang D Luo Y. Informing immunotherapy with multi-omics driven machine learning. NPJ Digit Med. (2024) 7:67. doi: 10.1038/s41746-024-01043-6, PMID: 38486092 PMC10940614

[120] Thalhamer T Dobias H Stepanoska T Pröll M Stutz H Dissertori O . Designing hypoallergenic derivatives for allergy treatment by means of in silico mutation and screening. J Allergy Clin Immunol. (2010) 125:e10. doi: 10.1016/j.jaci.2010.01.031 20371399

[121] Yu X-X Liu M-Q Li X-Y Zhang Y-H Tao B-J. Qualitative and quantitative prediction of food allergen epitopes based on machine learning combined with in vitro experimental validation. Food Chem. (2023) 405:134796. doi: 10.1016/j.foodchem.2022.134796, PMID: 36335724

[122] Ananya Panchariya DC Karthic A Singh SP Mani A Chawade A Kushwaha S. Vaccine design and development: exploring the interface with computational biology and AI. Int Rev Immunol. (2024) 43:361–80. doi: 10.1080/08830185.2024.2374546, PMID: 38982912

[123] Bai G Sun C Guo Z Wang Y Zeng X Su Y . Accelerating antibody discovery and design with artificial intelligence: recent advances and prospects. Semin Cancer Biol. (2023) 95:13–24. doi: 10.1016/j.semcancer.2023.06.005, PMID: 37355214

[124] Gangwal A Lavecchia A. Artificial intelligence in natural product drug discovery: current applications and future perspectives. J Med Chem. (2025) 68:3948–69. doi: 10.1021/acs.jmedchem.4c01257, PMID: 39916476 PMC11874025

[125] Sharma BK. AI managed hospital workforce. In: Chatterjee JM Sujatha R Saxena SK, editors. Role of artificial intelligence, telehealth, and telemedicine in medical virology. Singapore: Springer (2025).

[126] Gupta P Pandey MK. Role of AI for smart health diagnosis and treatment. In: Dey N Misra B Chakraborty S, editors. Smart medical imaging for diagnosis and treatment planning. Boca Raton, FL: Chapman and Hall/CRC (2024)

[127] Liu J Bao C Zhang J Han Z Fang H Lu H. Artificial intelligence with mass spectrometry-based multimodal molecular profiling methods for advancing therapeutic discovery of infectious diseases. Pharmacol Ther. (2024) 263:108712. doi: 10.1016/j.pharmthera.2024.108712 39241918

[128] Rajpurkar P Lungren MP. The current and future state of Ai interpretation of medical images. N Engl J Med. (2023) 388:1981–90. doi: 10.1056/NEJMra2301725, PMID: 37224199

[129] Esmaeilzadeh P Mirzaei T Dharanikota S. Patients’ perceptions toward human–artificial intelligence interaction in health care: experimental study. J Med Internet Res. (2021) 23:e25856. doi: 10.2196/25856, PMID: 34842535 PMC8663518

[130] Wang F Kaushal R Khullar D. Should health care demand interpretable artificial intelligence or accept “black box” medicine? Am Intern Med. (2020) 172:59–60. doi: 10.7326/M19-2548 31842204

[131] Tang PM Koopman J Mai KM De Cremer D Zhang JH Reynders P . No person is an island: unpacking the work and after-work consequences of interacting with artificial intelligence. J Appl Psychol. (2023) 108:1766–89. doi: 10.1037/apl0001103, PMID: 37307359

[132] Al-Zahrani AM. Unveiling the shadows: beyond the hype of AI in education. Heliyon. (2024) 10:e30696. doi: 10.1016/j.heliyon.2024.e30696, PMID: 38737255 PMC11087970

[133] Zhai C Wibowo S Li LD. The effects of over-reliance on Ai dialogue systems on students' cognitive abilities: a systematic review. Smart Learn Environ. (2024) 11:28. doi: 10.1186/s40561-024-00316-7, PMID: 40148911

